# Generation and characterization of OX40-ligand fusion protein that agonizes OX40 on T-Lymphocytes

**DOI:** 10.3389/fimmu.2024.1473815

**Published:** 2025-01-10

**Authors:** Ayaka Sato, Hodaka Nagai, Ayano Suzuki, Aya Ito, Shimpei Matsuyama, Nagito Shibui, Masashi Morita, Mari Hikosaka-Kuniishi, Naoto Ishii, Takanori So

**Affiliations:** ^1^ Laboratory of Molecular Cell Biology, Graduate School of Medicine and Pharmaceutical Sciences, University of Toyama, Toyama, Japan; ^2^ Department of Microbiology and Immunology, Tohoku University Graduate School of Medicine, Tohoku University, Sendai, Japan

**Keywords:** OX40L, OX40, co-stimulation, T cell, TNF superfamily, TNF receptor superfamily, agonist

## Abstract

OX40, a member of the tumor necrosis factor (TNF) receptor superfamily, is expressed on the surface of activated T cells. Upon interaction with its cognate ligand, OX40L, OX40 transmits costimulatory signals to antigen-primed T cells, promoting their activation, differentiation, and survival^—^processes essential for the establishment of adaptive immunity. Although the OX40-OX40L interaction has been extensively studied in the context of disease treatment, developing a substitute for the naturally expressed membrane-bound OX40L, particularly a multimerized OX40L trimers, that effectively regulates OX40-driven T cell responses remains a significant challenge. In this study, we successfully engineered soluble OX40L-fusion proteins capable of robustly activating OX40 on T cells. This was achieved by incorporating functional multimerization domains into the TNF homology domain of OX40L. These OX40L proteins bound to OX40, subsequently activated NF-κB signaling, and induced cytokine production by T cells *in vitro*. *In vivo*, mice treated with one of the OX40L-fusion proteins^—^comprising a single-chain OX40L trimer linked to the C-terminus of the human IgG1 Fc domain, forming a dimer of trimers^—^exhibited significantly enhanced clonal expansion of antigen-specific CD4^+^ T cells during the primary phase of the immune response. A comparable antibody-fusion single-chain TNF protein incorporating 4-1BBL, CD70 (CD27L), or GITRL in place of OX40L elicited similar *in vivo* T cell responses. Thus, we propose that optimizing the multimerization of OX40L proteins through innovative design strategies may facilitate the development of more effective agonists for targeted immunotherapies.

## Introduction

The tumor necrosis factor receptor (TNFR) family molecule OX40 (CD134, TNFRSF4) is induced on activated CD4^+^ and CD8^+^ T cells and transmits vital costimulatory signals to antigen-primed T cells. OX40 promotes T-cell activation, differentiation, and survival through interaction with its cognate OX40 ligand (OX40L, CD252, TNFSF4), which is expressed by activated professional antigen-presenting cells, including dendritic cells, macrophages, and B cells. The interaction between OX40 and OX40L augments the clonal expansion of effector and memory T cell populations and also plays an important role in regulatory T cell activity. For this reason, the OX40-OX40L system has been a promising target for immunotherapy (1–9).

T-cell-stimulating agonistic anti-OX40 antibodies and OX40L-fusion proteins deliver costimulatory signals that enhance the tumoricidal CD4^+^ and CD8^+^ T cells in preclinical models (10–15). Two OX40L agonist proteins, OX40L-IgG4-Fc (MEDI6383) (16) and PD-1-IgG4-OX40L (SL-279252) (17), have been evaluated in phase I clinical trials (18). Despite extensive efforts to develop biotherapeutics targeting OX40 for cancer, therapies with OX40 agonists have demonstrated only modest clinical efficacy (19). Therefore, fundamental analyses are still required to identify the types of agonistic agents that can effectively control OX40-driven T cell responses. The regulatory mechanisms of agonists are difficult to generalize. Additionally, it remains unclear which molecules could substitute for naturally expressed OX40L on the cell surface and outperform the activity of endogenous OX40L. Investigating OX40 agonism using various types of OX40 agonists could pave the way for the development of novel OX40-targeted immunotherapies.

The C-terminal TNF homology domain (THD) of OX40L assembles to form a membrane-bound homotrimer, which promotes the trimerization of OX40 expressed by T cells (20). Receptor oligomerization and clustering through interaction with cognate ligands are critical for the activation of TNFR signaling pathways (21, 22). An important question is how to create a potent OX40L molecule that can induce OX40 costimulatory signaling, leading to beneficial T cell responses. We previously generated a soluble OX40L protein possessing a collagenous trimerization domain derived from mannose-binding lectin (MBL) (23) and a soluble OX40L in a single-chain format connected to the C-terminus of the IgG Fc domain (24).

In this study, we prepared five different types of soluble OX40L-fusion proteins with MBL and/or Fc to evaluate the structural and functional relationships of OX40L in terms of OX40 agonism. These OX40L-fusion proteins possess different OX40L trimer moieties. We found that these OX40L-fusion proteins bound to OX40 and induced NF-κB signaling. Costimulatory signaling induced by these OX40L-fusion proteins significantly promoted the production of cytokines by CD4^+^ and CD8^+^ T cells *in vitro*. Administration of one of the OX40L-fusion proteins to mice immunized with an antigen significantly promoted the expansion of antigen-specific conventional CD4^+^ T cells producing effector cytokines. Other TNF family ligand-fusion proteins containing 4-1BBL, CD70, or GITRL, which share a similar structure, also significantly enhanced T cell responses. Our results demonstrate that TNFR agonism can be controlled through rational design of the molecular structure of TNF ligand proteins.

## Materials and methods

### Plasmids

Vectors containing cDNA encoding mouse *Ox40l* (*Tnfsf4*), mouse mannose-binding lectin 1 (*Mbl1*), mouse *4-1bbl* (*Tnfsf9*), mouse *Cd70*, mouse *Gitrl* (*Tnfsf18*), or mouse dihydrofolate reductase (*Dhfr*) were previously described (23–25). The MBL-OX40L gene (**Supplementary Figure S1A**) in the pCAGGS plasmid (GenBank accession number LT727518.1) was also previously described (23). The MBL-OX40L protein consists of an N-terminal PA-peptide tag (GVAMPGAEDDVV), a collagen-like domain of Mbl1 (^18^Ser-^126^Gly), and a C-terminal extracellular THD domain of Ox40l (^51^Ser-^198^Leu). The Fc-MBL-OX40L gene (**Supplementary Figure S1B**) was constructed by inserting the MBL-OX40L gene into the 3’ end of the human IgG1 Fc gene using In-Fusion cloning (638947, In-Fusion Snap Assembly Master Mix, Takara Bio, Shiga, Japan). The Fc-MBL-OX40L protein is composed of an N-terminal Fc (hinge, CH_2_, and CH_3_ domains) and a C-terminal MBL-OX40L. The gene for Fc-scOX40L (**Supplementary Figure S1C**) was previously reported (24). The genes for scOX40L-Fc (**Supplementary Figure S1D**) and MBL-scOX40L (**Supplementary Figure S1E**) were constructed using In-Fusion cloning. The genes for Fc-sc4-1BBL, Fc-scCD70, and Fc-scGITRL were previously reported (24). The pCAGGS plasmid encoding mouse Ox40-Fc was also previously described (23).

### Recombinant proteins

To produce recombinant proteins, the expression vector was transfected into DG44-CHO cells (*Dhfr* negative, CRL-9096, ATCC, Manassas, VA, U.S.A.), and a stable DG44-CHO cell clone was established. In brief, DG44-CHO cells were cultured in IMDM (098-06465, FUJIFILM Wako Pure Chemical Corporation, Osaka, Japan) containing 0.1 mM hypoxanthine, 0.016 mM thymidine, 0.002 mM methotrexate (MTX), 50 µM 2-mercaptoethanol (2-ME), 2 mM L-Alanyl-L-Glutamine, 10% FCS, 100 U/mL penicillin, and 100 µg/mL streptomycin. The cells were then transfected with a pcDNA3.1 vector encoding both *Dhfr* gene and neomycin resistance gene, and concurrently with the pCAGGS vector encoding MBL-OX40L, Fc-MBL-OX40L, Fc-scOX40L, scOX40L-Fc, or MBL-scOX40L, at a 1:10 ratio. The transfected DG44-CHO cells were cultured in complete IMDM supplemented with 0.5 mg/mL G418, without hypoxanthine, thymidine, and MTX. To induce a high degree of gene amplification, the MTX concentration was increased stepwise from 50 to 800 nM, and individual clones were isolated from heterogeneous cell pools by limiting dilution. To select cell clones with the highest productivity, the expression level of recombinant protein in the culture supernatant was analyzed by using a sandwich enzyme-linked immunosorbent assay (ELISA). Selected DG44-CHO cell clones were cultured either in IMDM containing 2% FCS or BalanCD™ CHO GROWTH A/BalanCD™ CHO FEED4 (554-34287, 530-94421, FUJIFILM).

For the purification of Fc-MBL-OX40L, Fc-scOX40L, and scOX40L-Fc, the culture supernatant containing the respective recombinant OX40L proteins was applied to a HiTrap™ rProtein A FF column (17507901, Cytiva, Tokyo, Japan). After rinsing the column with phosphate-buffered saline (PBS), the bound Fc protein was eluted with elution buffer (80 mM glycine-HCl, 0.8 M arginine, pH 3.0), and the protein fractions were immediately neutralized with 1 M Tris-HCl (pH 9.0). MBL-scOX40L, possessing a His-tag, was purified using a HisTrap™ excel column (17371205, Cytiva). The column was washed with wash buffer (20 mM sodium phosphate, 0.5 M NaCl, 30 mM imidazole, pH 7.4) and eluted with elution buffer (20 mM sodium phosphate, 0.5 M NaCl, 500 mM imidazole, 0.8 M L-arginine, pH 7.4). MBL-OX40L and MBL-scOX40L containing a PA tag were absorbed onto Anti PA tag Antibody Beads (012-25841, FUJIFILM). The beads were washed with PBS and eluted with elution buffer (0.1 M glycine-HCl, 1 M arginine, 2 M MgCl_2_·6H_2_O, pH 3.0). The eluate was dialyzed against PBS, concentrated using an Amicon Ultra-4 (UFC801008, Merck MilliporeSigma, Burlington, Massachusetts, U.S.A.), and filtered with Millex-GP (0.22 µm, SLGPR33RS, Merck). The total protein concentration was determined using a bicinchoninic acid (BCA) assay (297-73101, FUJIFILM).

### ELISA

To detect the binding between OX40 and OX40L, purified OX40L proteins were added to ELISA plates (439454, Thermo Fisher, Waltham, MA, U.S.A.) pre-coated with 0.4 µg/mL of OX40-Fc (23, 26). OX40L protein was detected with anti-PA tag IgG (NZ-1, 012-25863, FUJIFILM) and peroxidase-conjugated AffiniPure Goat Anti-Rat IgG (H+L) (112-035-167, Jackson ImmunoResearch, West Grove, PA, U.S.A.).

The following reagents were used for the measurement of cytokines from T cells: anti-IL-2 (JES6-1A12, 503701, BioLegend, San Diego, CA, U.S.A.) capture antibody, biotin-anti-IL-2 (JES6-5H4, 503803, BioLegend) detection antibody, anti-IFN-γ (R4-6A2, 505702, BioLegend) capture antibody, biotin-anti-IFN-γ (XMG1.2, 505804, BioLegend) detection antibody, anti-IL-4 (11B11, 504101, BioLegend) capture antibody, biotin-anti-IL-4 (BVD6-24G2, 504201, BioLegend) detection antibody, anti-IL-17A (TC11-18H10.1, 506901, BioLegend) capture antibody, biotin-anti-IL-17A (TC11-8H4, 507001, BioLegend) detection antibody, HRP streptavidin (405210, BioLegend), and 3,3’,5,5’-tetramethylbenzidine (TMB, 421101, BioLegend). The absorbance was measured at 450 nm or 620 nm using a FilterMax F5 (Molecular Devices, San Jose, CA, U.S.A.).

### Flow cytometry

An OX40^+^ T cell hybridoma was previously established and used to evaluate OX40 activity in T cells (23–25, 27, 28). To test the binding activity of OX40L proteins to cell-associated OX40 proteins, the T cell hybridoma was first incubated with the respective OX40L proteins, followed by incubation with anti-PA tag antibody, and then with goat anti-rat IgG2a-FITC (MRG2a-83, 407506, BioLegend).

To perform the activation-induced marker (AIM) assay, a valuable method to detect antigen-specific CD4^+^ T cells (29, 30), draining lymph node (dLN) cells were initially incubated with IgG from rat serum (14131, Merck) to block nonspecific binding. The following antibodies were used for cell-surface staining: PE/Cyanine7 anti-mouse CD4 (GK1.5, 100421, BioLegend), Alexa Fluor^®^ 488 anti-mouse CD25 (PC61, 102018, BioLegend), and APC anti-mouse CD134 (OX-40) (OX-86, 119413, BioLegend). For Foxp3 staining, cells were fixed and permeabilized with the eBioscience™ Foxp3/Transcription Factor Staining Buffer Set (00-5523-00, Thermo Fisher), then stained with PE Anti-Mo/Ra FOXP3, eBioscience™ (FJK-16s, 12-5773-08, Thermo Fisher). Data were acquired on a FACS Canto II or a Celesta (BD Biosciences, Franklin Lakes, NJ, U.S.A.) and analyzed with BD FACS Diva software version 9 or Flowing software version 2 (Turku Bioscience, Turku, Finland, http://flowingsoftware.btk.fi/).

### Immunoblot analysis

Recombinant OX40L proteins were mixed with 4× Laemmli sample buffer (240 mM Tris-HCl, pH 6.8, 40% glycerol, 8% sodium dodecyl sulfate (SDS), 0.1% bromophenol blue) with or without 4% 2-ME and incubated at 60-100°C for 1 to 5 minutes. Reducing or non-reducing samples were separated by SDS-polyacrylamide gel electrophoresis (PAGE), transferred onto polyvinylidene difluoride (PVDF) membranes (034-25663, FUJIFILM), and analyzed by immunoblotting with anti-PA tag antibody and peroxidase-conjugated AffiniPure Goat Anti-Rat IgG (H+L).

To detect the expression levels of IκBα and β-actin proteins, the OX40^+^ T cell hybridoma was lysed for 30 minutes in ice-cold radioimmunoprecipitation assay (RIPA) buffer (20 mM Tris-HCl, pH 7.5, 150 mM NaCl, 2 mM EDTA, 1% Nonidet P-40 (NP-40), 50 mM NaF, 1 mM Na_3_VO_4_, 1% sodium deoxycholate, and 0.1% SDS) with a protease inhibitor mixture (P8340, Merck). Insoluble debris was removed by centrifugation at 15,000×g for 10 minutes. The clear lysate was reduced with 4× Laemmli sample buffer for 5 minitues at 100°C. Samples were separated by SDS-PAGE, transferred onto PVDF membranes, and analyzed by immunoblotting with anti-IκBα (L35A5, 4814, Cell Signaling Technology, Danvers, MA, U.S.A.), anti-β-actin (C4, MAB1501, Merck) or anti-β-tubulin (TUB2.1, T4026, Merk), followed by goat anti-mouse IgG (H+L chain) pAb-HRP (330, MEDICAL & BIOLOGICAL LABORATORIES CO., LTD., Tokyo, Japan). The reaction was visualized with a chemiluminescence detection system using Immobilon Classico Western HRP substrate (WBLUC0500, Merck) and LAS-4000 mini (FUJIFILM).

### T cell stimulation assay

C57BL/6 mice (Japan SLC, Shizuoka, Japan) were bred under specific pathogen-free conditions. Animal experimental protocols were approved by the Animal Care and Use Committee of the University of Toyama (Approved numbers: A2024PHA-02 and A2024PHA-03) and conducted in accordance with the Institutional Animal Experiment Handling Rules of the University of Toyama. CD4^+^ and CD8^+^ T cells were purified from the spleens of C57BL/6 mice using CD4 (L3T4) microbeads (130-117-043, Miltenyi Biotec, Bergish Gladbach, Germany) and CD8 (Ly-2) microbeads (130-117-044), respectively. T cells were cultured in RPMI medium with 10% FCS, 2 mM L-Alanyl-L-Glutamine, 100 U/mL penicillin, 100 µg/mL streptomycin, and 50 µM 2-ME. T cells were plated in 200 µL volumes in 96-well flat-bottom culture plates precoated with 10 µg/mL of anti-CD3ε antibody (low endotoxin, azide-free, 145-2C11, 100340, Biolegend) in triplicate with increasing concentrations of respective OX40L proteins. Culture supernatants were harvested at 72 hours for cytokine ELISA.

### Immunization

Groups of C57BL/6 mice from both male and female mice aged between 5 and 15 weeks were immunized subcutaneously at the footpad with 100 µg of 2,4,6-trinitrophenyl-conjugated keyhole limpet hemocyanin (TNP-KLH) emulsified in complete Freund’s adjuvant (CFA) (F5881, Merck) on day 0. Recombinant proteins in PBS were injected intraperitoneally on days 1 and 3. On day 7, pooled dLN cells were plated in 200 µL volumes in 96-well plates in triplicate in the presence or absence of KLH. Cells were harvested at 18 hours for the AIM assay. Culture supernatants were harvested at 72 hours for cytokine ELISA. Cell viability on day 3 was assessed with a 3-(4,5-dimethylthiazol-2-yl)-2,5-diphenyl-tetrazolium (MTT) (M2128, Merck) assay.

### Statistical analysis

Statistical significance was evaluated using either the Tukey-Kramer or Dunnett’s multiple comparison test in JMP Pro version 17.0.0 (SAS Institute, Cary, NC, U.S.A.). A *p*-value of less than 0.05 was considered statistically significant.

## Results

### Structure of OX40L-fusion proteins

**Figure 1:**
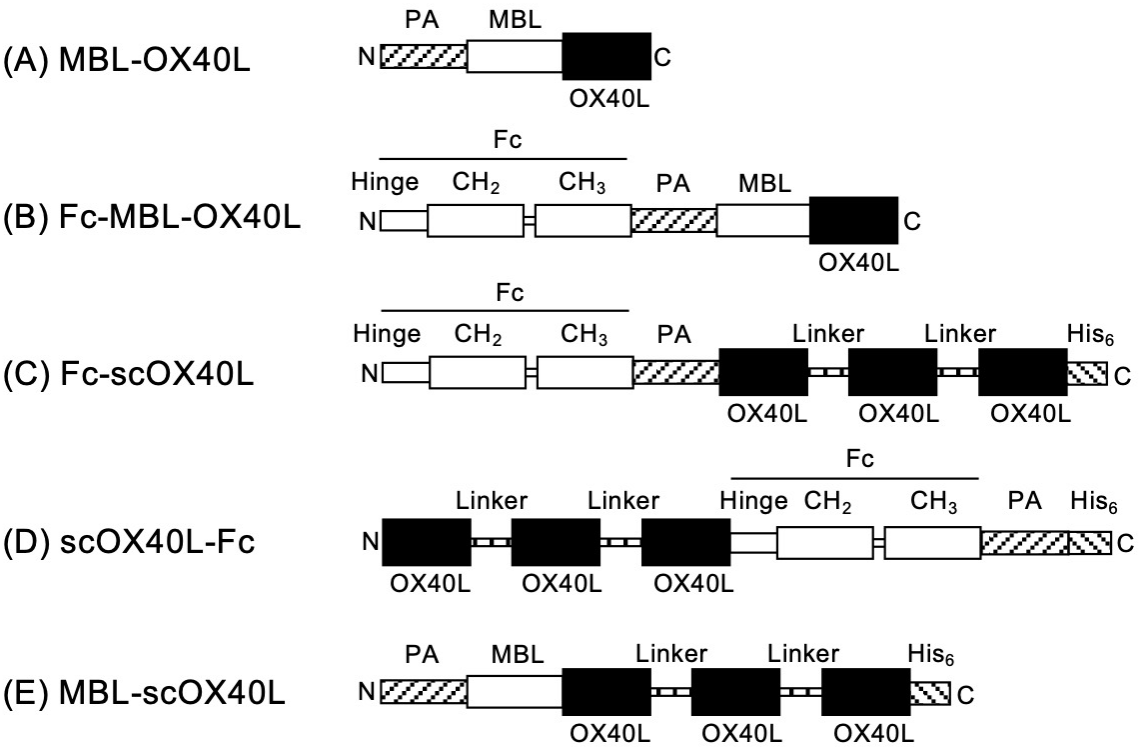
Schematic representation of OX40L-fusion proteins. The domain structures of the respective OX40L-fusion proteins are as follows: **(A)** MBL-OX40L [PA peptide tag (PA, GVAMPGAEDDVV), collagen-like domain of mannose-binding lectin (MBL, ^18^Ser-^126^Gly), and extracellular TNF homology domain of OX40L (OX40L, ^51^Ser-^198^Leu)]; **(B)** Fc-MBL-OX40L [Fc (hinge, CH_2_, and CH_3_ domains of human IgG1), PA, MBL, and OX40L]; **(C)** Fc-scOX40L [Fc, PA, OX40L, GGGSGGG peptide linker (Linker), OX40L, Linker, OX40L, and histidine peptide tag (His_6_, HHHHHH)]; **(D)** scOX40L-Fc [OX40L, Linker, OX40L, Linker, OX40L, Fc, PA, and His_6_]; **(E)** MBL-scOX40L [PA, MBL, OX40L, Linker, OX40L, Linker, OX40L, and His_6_]. The nucleotide sequences of the OX40L-fusion proteins are shown in **Supplementary Figure S1**.

Highly clustered trimeric OX40L molecules, expressed by antigen-presenting cells, exhibit potent agonistic functions and are likely the natural source of costimulatory activity for OX40 on T cells during physiologically relevant immune responses. Identifying a substitute for naturally expressed OX40L on the cell surface remains a significant challenge. We hypothesized that highly multimerized OX40L trimers could serve as potent agonists for OX40. To investigate how the structure of the OX40L protein influences its agonistic activity toward OX40, we prepared five different soluble OX40L-fusion proteins, as illustrated in **Figure 1**. The DNA nucleotide sequences corresponding to these OX40L proteins are provided in **Supplementary Figure S1**. The structure of MBL-OX40L protein consists of a PA peptide tag (PA), a collagen-like domain derived from mannose-binding lectin (MBL), and the THD of OX40L (OX40L), from the N-terminus to the C-terminus (**Figure 1A**) (23). The attachment of the Fc domain of IgG1 (Fc) to MBL induces the formation of oligomerized trimeric structures of CD40L, which efficiently stimulates CD40 on B cells (31). Consequently, we prepared Fc-MBL-OX40L, comprising Fc, PA, MBL, and OX40L (**Figure 1B**). The structure of Fc-scOX40L is composed of Fc, PA, OX40L, a GGGSGGG peptide linker (Linker), OX40L, Linker, OX40L, and a histidine peptide tag (His_6_) (**Figure 1C**) (24). The structure of scOX40L-Fc contains OX40L, Linker, OX40L, Linker, OX40L, Fc, PA, and His_6_ (**Figure 1D**). The structure of MBL-scOX40L includes PA, MBL, OX40, Linker, OX40L, Linker, OX40L, and His_6_ (**Figure 1E**). The structural moieties PA, His, and Fc were employed for the detection and purification of the protein. The moieties MBL and Fc were utilized to facilitate the formation of a trimer and a dimer, respectively. The tandem repeats of three OX40Ls, scOX40L, were designed to organize the formation of an active OX40L trimer.

**Figure 2:**
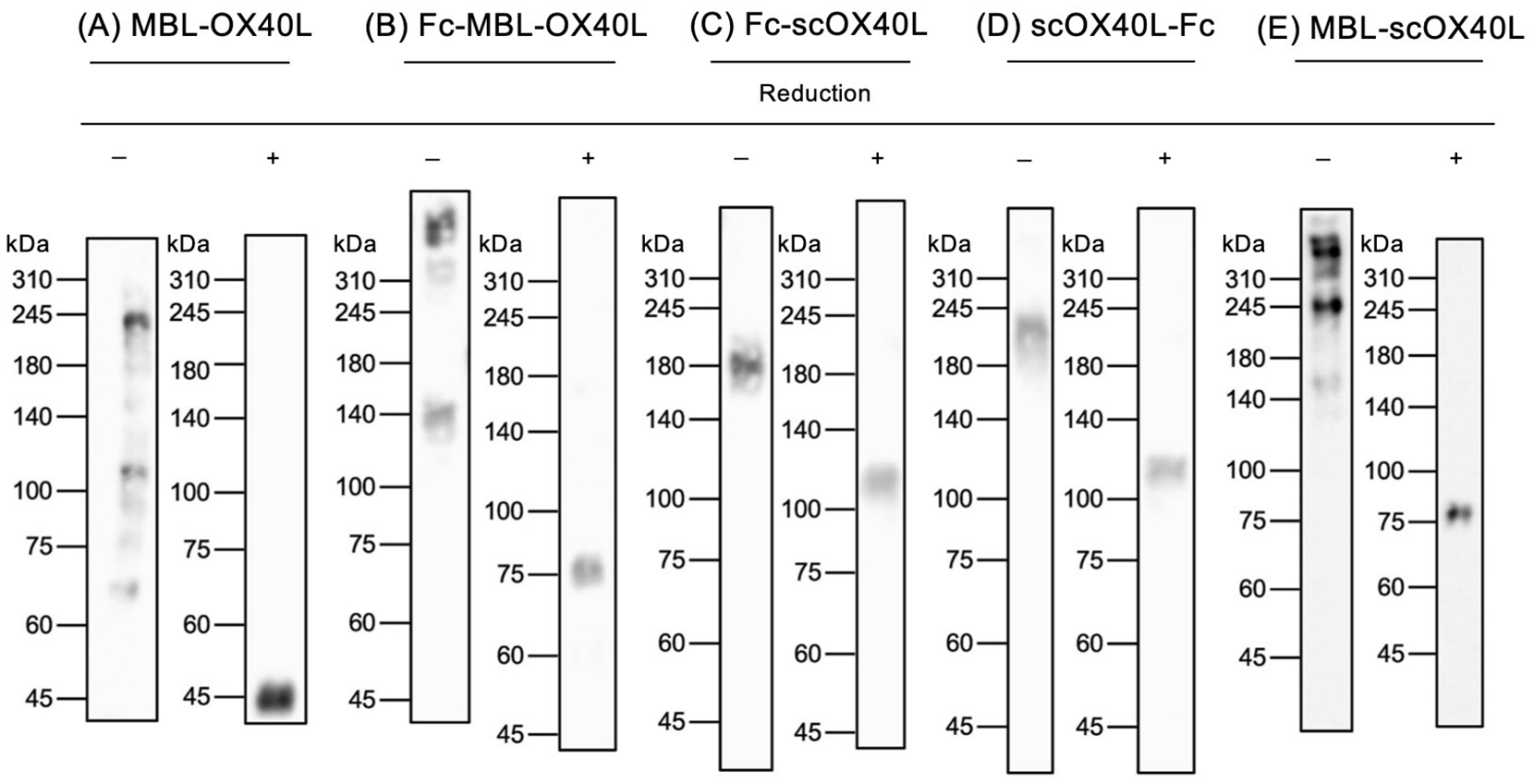
Immunoblot analysis of OX40L-fusion proteins. Five different OX40L-fusion proteins **(A-E)** were analyzed by immunoblotting using anti-PA tag antibody under non-heated/non-reducing (-) and heated/reducing (+) conditions. The migration positions of molecular weight markers (kDa) are shown on the left side of each gel. The expected molecular weights of the reduced OX40L-fusion proteins, based on their amino acid sequences, are as follows: **(A)** MBL-OX40L (29 kDa); **(B)** Fc-MBL-OX40L (55 kDa); **(C)** Fc-scOX40L (79 kDa); **(D)** scOX40L-Fc (72 kDa); and **(E)** MBL-scOX40L (64 kDa). The discrepancy between the observed molecular masses and the calculated masses is suggested to be derived from glycosylation in OX40L (N91, UniProt P43488), MBL (K80 and K83, UniProt P39039), and Fc (N299, UniProt P0DOX5) proteins.

MBL-OX40L primarily displayed dimer, trimer, and hexamer structures based on molecular weight as observed in immunoblotting and SDS-PAGE, suggesting the intrinsic role of the collagenous trimerization domain of mannose-binding lectin in forming multimerized structures (32–34). In contrast, it appeared as a monomer after boiling and reduction in Laemmli’s buffer (**Figure 2A**; **Supplementary Figure S2A**) (23). Fc-MBL-OX40L showed dimer, hexamer, and higher-order multimer structures (**Figure 2B**; **Supplementary Figure S2B**). Fc-scOX40L and scOX40L-Fc exhibited a dimer structure (**Figures 2C, D**; **Supplementary Figures S2C, D**). MBL-scOX40L formed dimer, trimer, and multimeric structures (**Figure 2E**; **Supplementary Figure S2E**). These electrophoretic analyses suggest that MBL-OX40L, Fc-MBL-OX40L, and MBL-scOX40L containing MBL form multimeric conformations, whereas Fc-scOX40L and scOX40L-Fc form dimeric structures.

### OX40-binding activity of OX40L-fusion proteins

**Figure 3:**
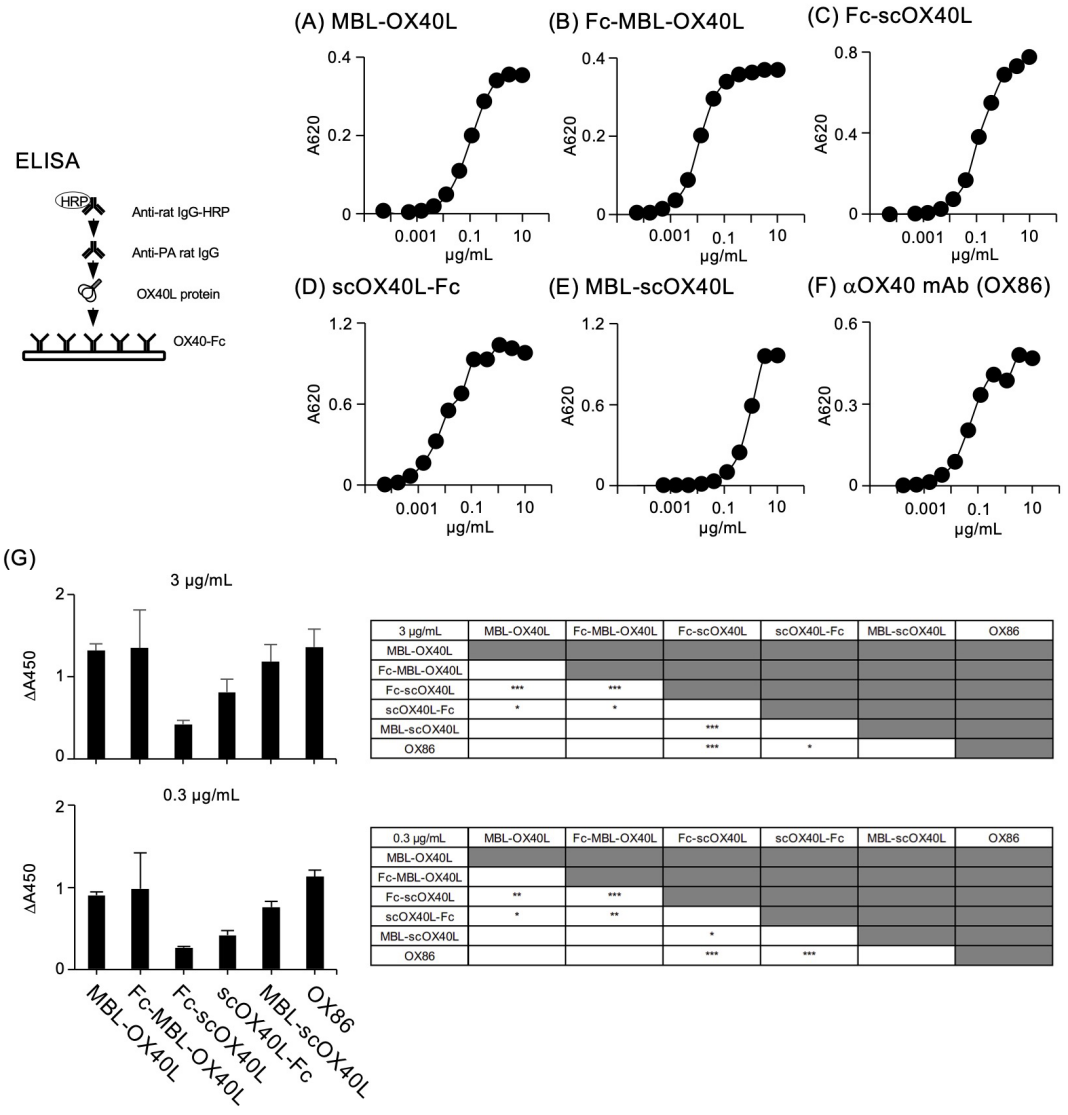
Binding of OX40L-fusion proteins to OX40-Fc, as evaluated by ELISA. Serially diluted OX40L-fusion proteins, including MBL-OX40L **(A)**, Fc-MBL-OX40L **(B)**, Fc-scOX40L **(C)**, scOX40L-Fc **(D)**, and MBL-scOX40L **(E)**, were added to ELISA wells precoated with OX40-Fc, and binding was detected using anti-PA rat IgG and anti-rat IgG-HRP. Anti-OX40 antibody (OX86) **(F)** was used as a positive control. Data are from one experiment that is representative of at least two independent experiments with similar results. **(G)** Comparison of the binding activity of OX40L-fusion proteins and OX86 toward OX40-Fc, evaluated by ELISA. Respective OX40L-fusion proteins and OX86 proteins at concentrations of 3 µg/mL or 0.3 µg/mL were used to detect binding. The background absorbance was subtracted from the absorbances of the corresponding samples (n = 4) (ΔA450). Similar results were obtained at 0.03 µg/mL of the respective proteins. *p < 0.05, **p < 0.01, and ***p < 0.001 indicate statistically significant differences among the groups as determined by the Tukey-Kramer test.

**Figure 4:**
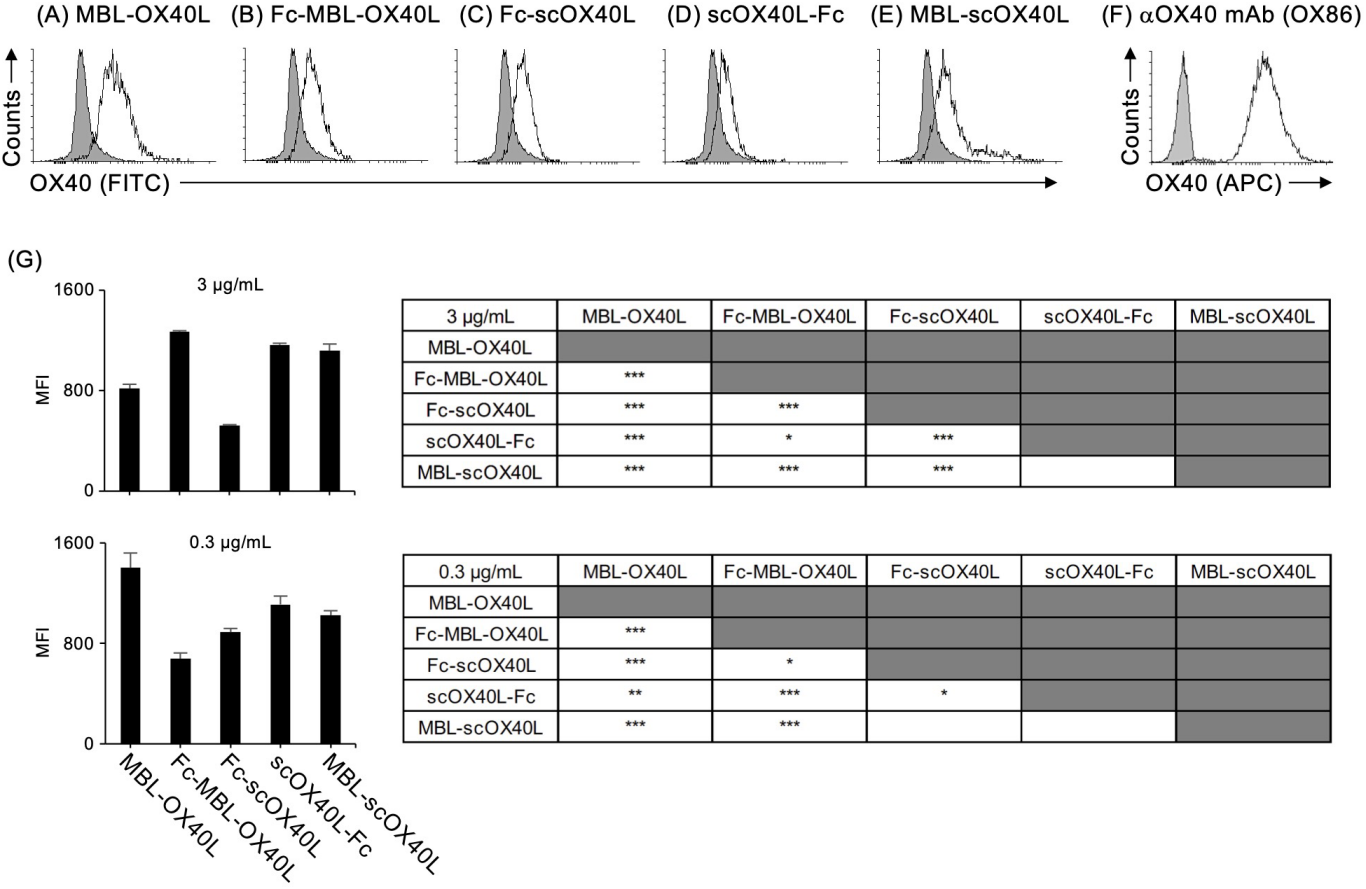
Binding activity of OX40L-fusion proteins to cell surface OX40, as evaluated by flow cytometry. The OX40-expressing T cell hybridoma was stained with the respective OX40L-fusion proteins, MBL-OX40L **(A)**, Fc-MBL-OX40L **(B)**, Fc-scOX40L **(C)**, scOX40L-Fc **(D)**, and MBL-scOX40L **(E)**. The OX40L-OX40 interaction was detected using anti-PA rat IgG and anti-rat IgG-FITC. Anti-OX40 antibody (OX86) **(F)** was used as a positive staining control. The negative staining control is shown as a shaded histogram. Data are from one experiment representative of at least two independent experiments with similar results. **(G)** Comparison of the binding activity of OX40L-fusion proteins to cell surface OX40, evaluated by flow cytometry and expressed as mean fluorescent intensity (MFI). OX40L-fusion proteins at concentrations of 3 µg/mL or 0.3 µg/mL were used to assess binding (n = 3). *p < 0.05, **p < 0.01, and ***p < 0.001 indicate statistically significant differences among the groups as determined by the Tukey-Kramer test.

Next, to confirm the binding activity of these OX40L proteins to OX40, each OX40L protein was serially diluted and added to the wells of a microtiter ELISA plate pre-adsorbed with OX40-Fc. Our previous studies demonstrated that MBL-OX40L and Fc-scOX40L specifically interact with OX40 (23, 24). Consistent with previous results, all five OX40L proteins and the control anti-OX40 monoclonal antibody (OX86) bound to OX40-Fc in a dose-dependent manner (**Figures 3A-F**) and exhibited different binding activities (**Figure 3G**). The OX40L moieties of these proteins did not cross-react with 4-1BB, CD27, or GITR (23, 24). Additionally, these five OX40L proteins interacted differentially with the cell surface OX40 expressed by the T cell hybridoma (**Figures 4A-G**; **Supplementary Figures S3A-E**). These results demonstrate that the OX40L-fusion proteins used in this study specifically bind to OX40.

### Agonistic activity of OX40L-fusion proteins

**Figure 5:**
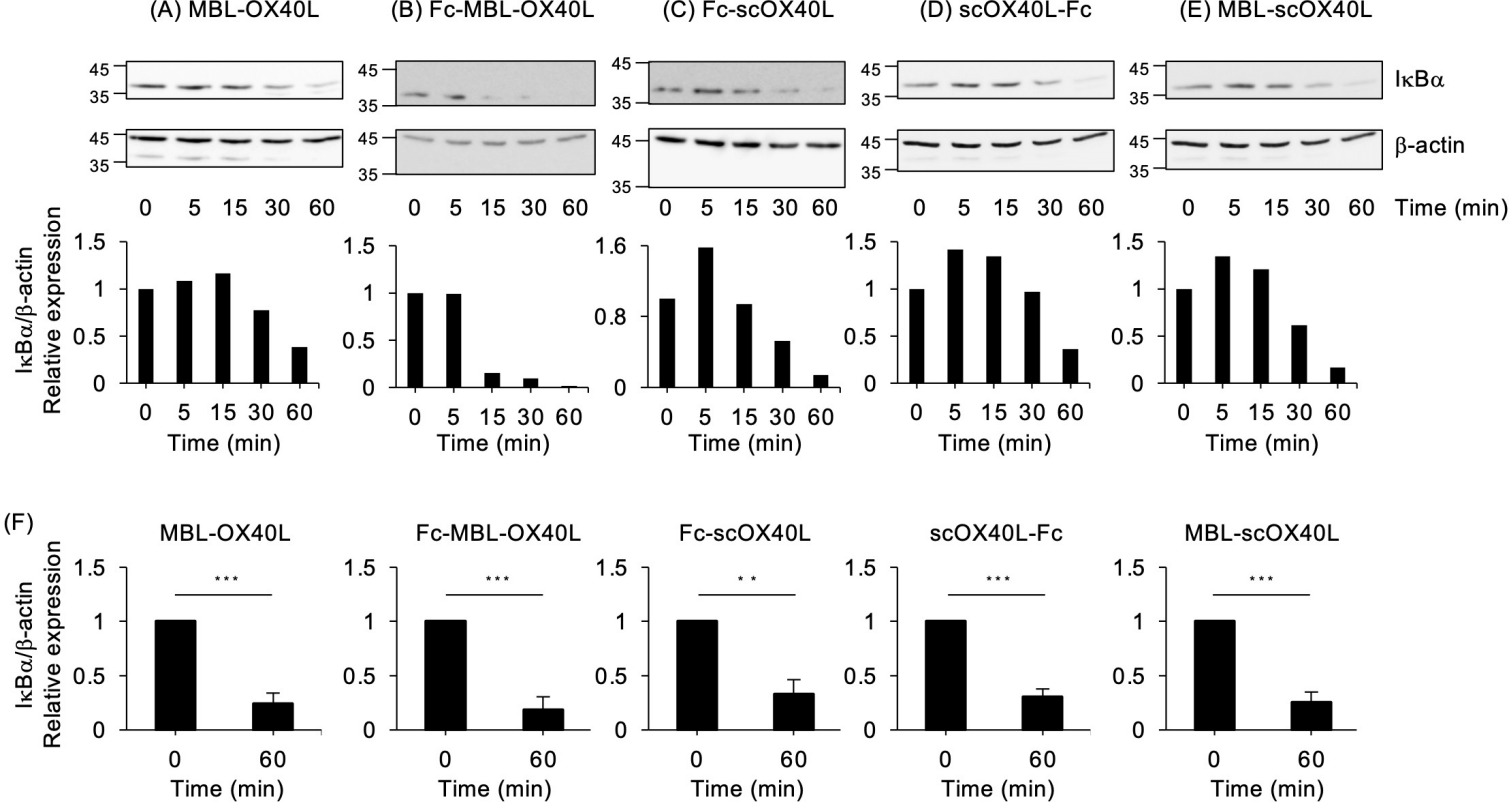
NF-κB activation mediated by OX40L-fusion proteins, as evaluated by immunoblotting. The OX40-expressing T cell hybridoma was incubated with 30 µg/mL of the respective OX40L-fusion proteins, MBL-OX40L **(A)**, Fc-MBL-OX40L **(B)**, Fc-scOX40L **(C)**, scOX40L-Fc **(D)**, and MBL-scOX40L **(E)**, for the indicated times. The cell lysate, equivalent to 4×10^4^ cells per well, was applied to an SDS-PAGE gel for electrophoresis, followed by transfer to a PVDF membrane. The protein expression of IκBα and β-actin was evaluated by immunoblotting (upper). The densitometric ratio of the IκBα band to the corresponding β-actin is shown (lower). Data are from one experiment representative of at least two independent experiments with similar results. **(F)** The densitometric ratio of IkBα band to the corresponding β-actin in OX40-exprerssing T cell hybridoma stimulated with the respective OX40L proteins for 60 min. Data are from three independent experiments. **p < 0.01 and ***p < 0.001 indicate statistically significant differences between the stimulated and unstimulated conditions, as determined by the Student’s *t*-test test.

OX40 activates the classical NF-κB pathway (27, 35). To evaluate the functional activity of OX40L-fusion proteins in terms of NF-κB activation, the T cell hybridoma used in the experiments shown in **Figure 4**; **Supplementary Figure S3** was stimulated with the respective OX40L-fusion proteins. As demonstrated in **Figure 5**; **Supplementary Figure S4**, all five OX40L-fusion proteins induced the degradation of IκBα, a hallmark of classical NF-κB pathway activation. The anti-OX40 agonistic antibody (OX86) did not efficiently promote the degradation of IκBα (**Supplementary Figure S5**), indicating that OX40L proteins possess an advantage in activating the NF-κB pathway.

**Figure 6:**
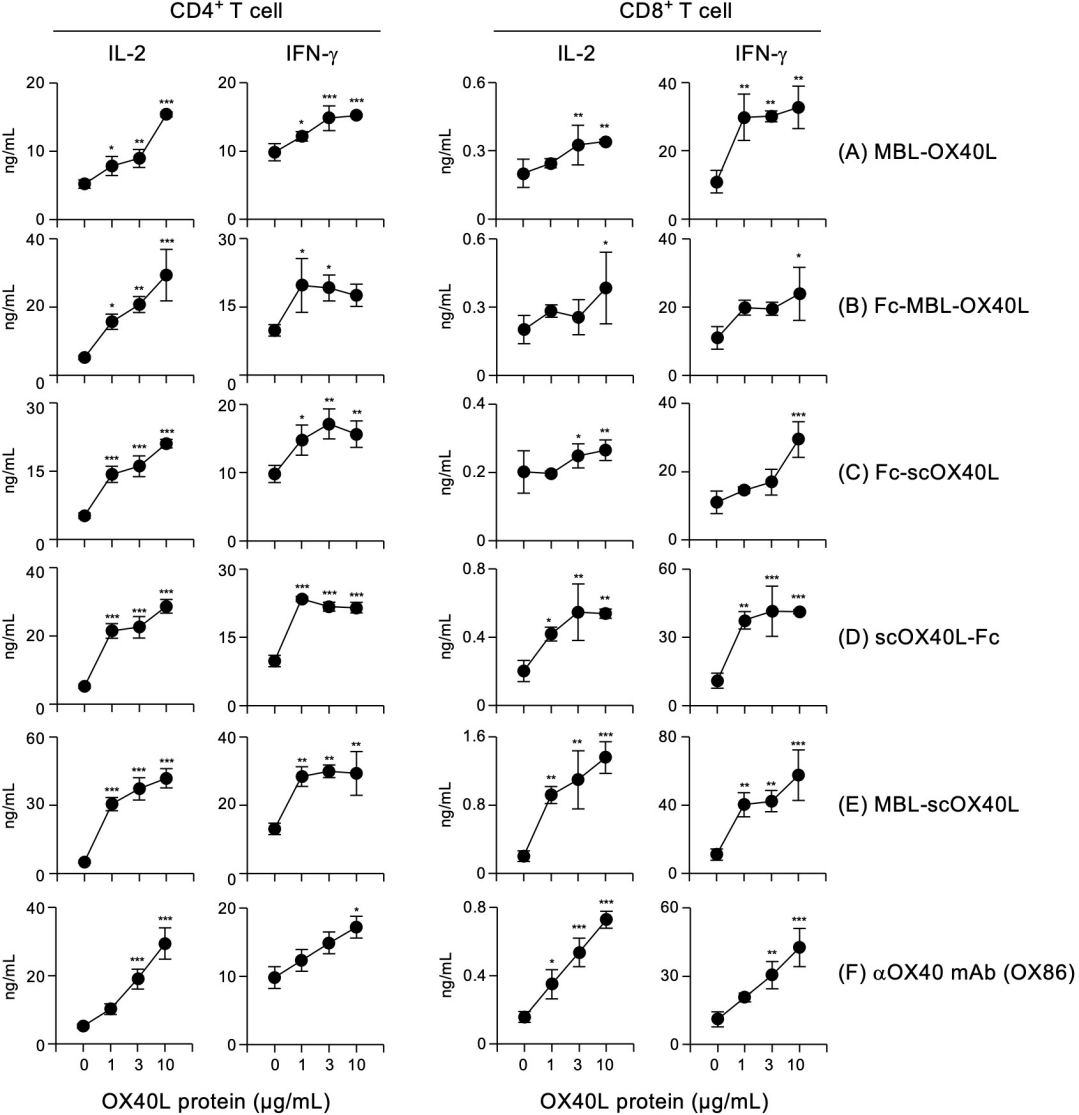
Dose-dependency of OX40L-fusion proteins for the induction of IL-2 and IFN-g from CD4^+^ and CD8^+^ T cells. CD4^+^ (left) or CD8^+^ (right) T cells (5×10^4^ cells/well), purified from the spleens of C57BL/6 mice, were cultured in 96-well flat-bottom plates precoated with 10 µg/mL anti-CD3 antibody, with or without the indicated concentrations of the respective OX40L-fusion proteins, MBL-OX40L **(A)**, Fc-MBL-OX40L **(B)**, Fc-scOX40L **(C)**, scOX40L-Fc **(D)**, MBL-scOX40L **(E)**, and anti-OX40 agonistic monoclonal antibody (OX86) **(F)** for 3 days. The concentrations of IL-2 and IFN-g in the culture supernatants were determined by ELISA. Data are presented as the mean ± standard deviation (n = 3) from one experiment representative of at least two independent experiments with similar results. *p < 0.05, **p < 0.01, and ***p < 0.001 indicate significant differences between conditions with and without OX40L stimulation (Dunnett’s test).

Signals through OX40 promote TCR/CD3 signals and enhance cytokine production from T cells. To evaluate the costimulatory activity of OX40L-fusion proteins, murine splenic CD4^+^ and CD8^+^ T cells were cultured with plate-immobilized anti-CD3 agonistic antibody and increasing concentrations of the respective soluble OX40L-fusion proteins. Since OX40 is highly upregulated on activated T cells, microwells were coated with a relatively higher concentration of anti-CD3 antibody (10 µg/mL). Lower concentrations of anti-CD3 did not exhibit productive cytokine responses (24). All five OX40L-fusion proteins and the control anti-OX40 agonistic antibody (OX86) significantly increased the production of IL-2 and IFN-γ from CD4^+^ and CD8^+^ T cells (**Figures 6A-F**). Collectively, these results demonstrate that all OX40L-fusion proteins have significant costimulatory activity on T cells *in vitro*.

### Antigen-specific T cell responses induced by OX40L-fusion proteins and other TNF ligand-fusion proteins

To demonstrate the agonistic function of OX40L-fusion proteins *in vivo*, mice were immunized with TNP-KLH and CFA on day 0, and then injected with Fc-MBL-OX40L (B), Fc-scOX40L (C), or scOX40L-Fc (D) on days 1 and 3 to analyze antigen-specific CD4^+^ T cell responses. We selected these OX40L-fusion proteins because the IgG1 Fc domain moiety may enhance the overall *in vivo* stability of the OX40L-fusion proteins, thereby providing potent agonistic activity. On day 7, dLN cells were cultured with or without KLH. Anti-OX40 agonistic antibody (OX86) and phosphate-buffered saline (PBS) were administered as positive and negative controls, respectively.

**Figure 7:**
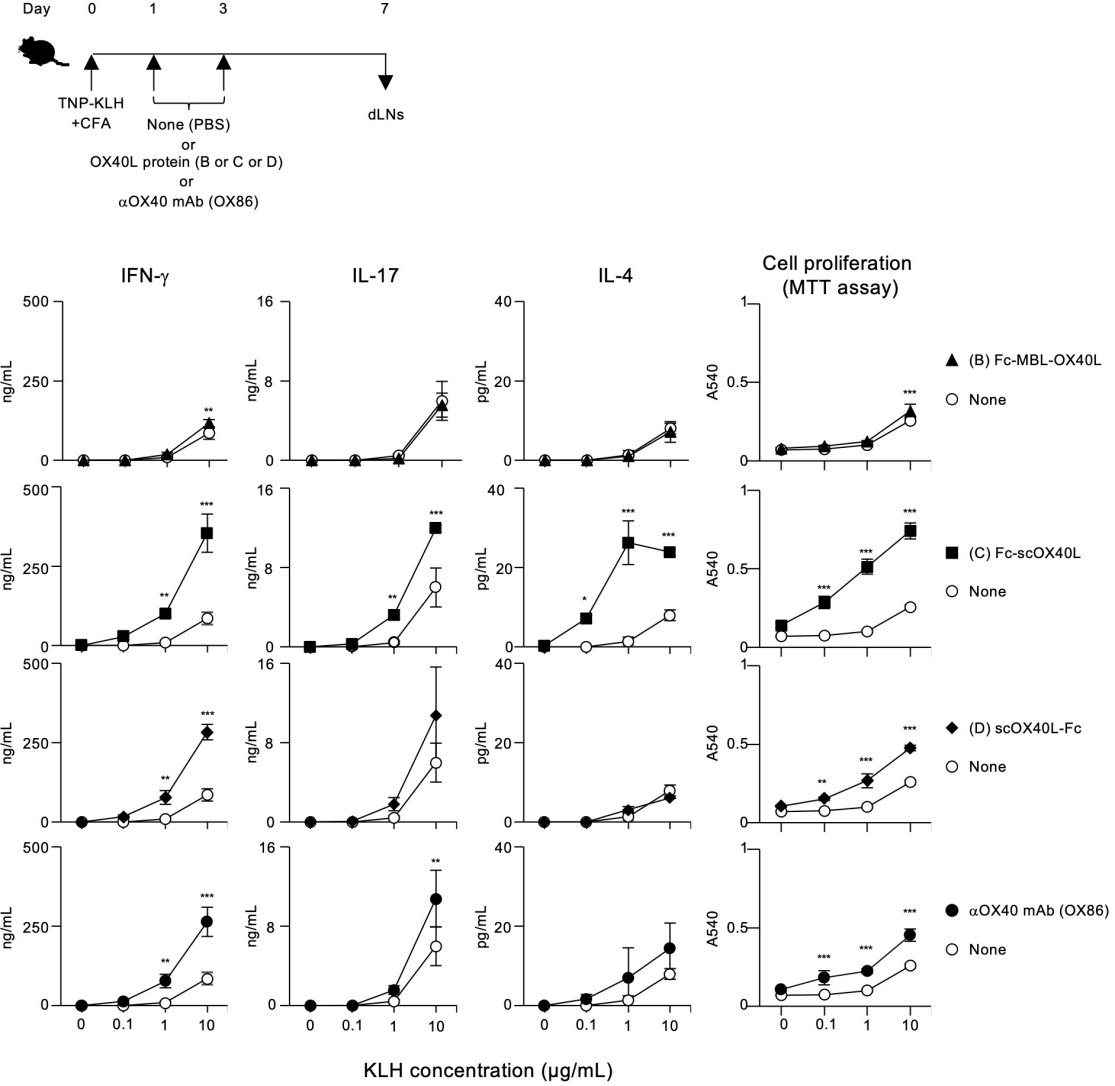
Enhanced antigen-specific effector T cell responses mediated by OX40L-fusion proteins. The experimental schedule for the induction of antigen-specific T cell responses is shown (upper). C57BL/6 mice were subcutaneously immunized with 100 µg of TNP-KLH emulsified in CFA on day 0, and then intraperitoneally injected twice with 20 µg of the respective OX40L-fusion proteins, Fc-MBL-OX40L, Fc-scOX40L, and scOX40L-Fc, on days 1 and 3. Anti-OX40 antibody (OX86) was used as a positive control. Popliteal draining lymph nodes (dLNs) were harvested on day 7. Pooled dLN cells from three mice per group were cultured with the indicated concentrations of KLH for 3 days. Cell proliferation was evaluated using the MTT assay. Cytokine concentrations were determined by ELISA. Data are presented as the mean ± standard deviation (n = 3) from one experiment representative of at least two independent experiments with similar results. *p < 0.05, **p < 0.01, and ***p < 0.001 indicate significant differences between the OX40L-injected group and the control (PBS) group at the respective antigen concentrations (Tukey-Kramer test).

Interestingly, Fc-scOX40L (C) elicited stronger antigen-specific T cell responses than Fc-MBL-OX40L (B) and scOX40L-Fc (D), as assessed by antigen recall responses of dLN cells, in terms of IFN-γ, IL-17, and IL-4 production and proliferation (**Figure 7**; **Supplementary Figure S6**). Both scOX40L-Fc (D) and anti-OX40 agonistic antibody (OX86) also triggered significantly higher T cell responses compared to the None (PBS) control group, although these responses were comparatively weaker (**Figure 7**). Unexpectedly, Fc-MBL-OX40L (B) failed to effectively stimulate *in vivo* T cell responses, with levels comparable to those observed in the None (PBS) control group (**Figure 7**; **Supplementary Figure S6**). These findings indicate that oligomerization of OX40L with MBL and IgG1 Fc domains, as well as its stabilization through fusion with IgG1 Fc domain, may not be sufficient to produce a more effective OX40 agonist.

**Figure 8:**
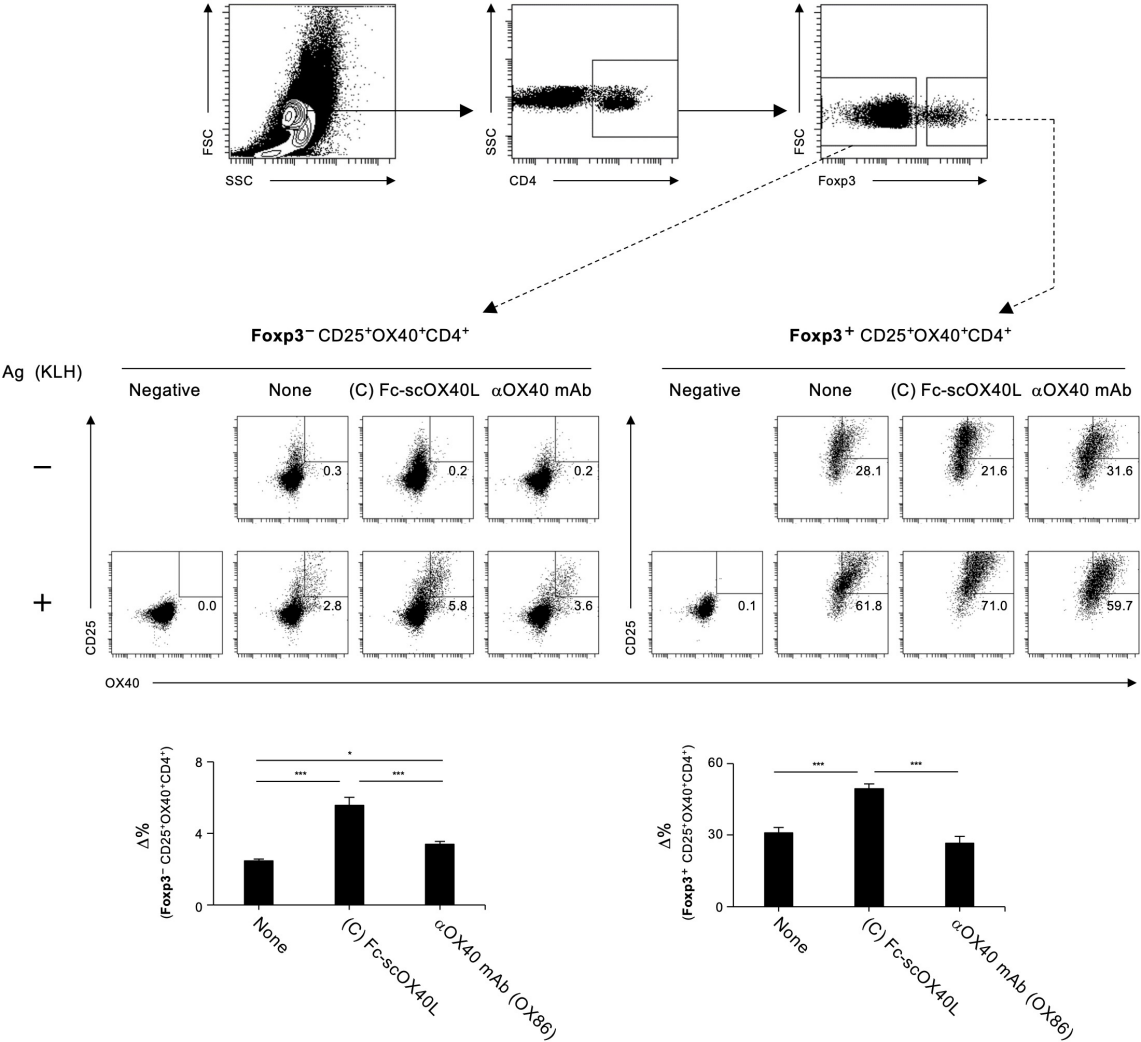
Quantification of antigen-specific CD4^+^ T cell responses mediated by OX40L-fusion proteins using the AIM assay. The gating strategy for identifying antigen-responsive, activated OX40^+^CD25^+^ cells within CD4^+^Foxp3^-^ and CD4^+^Foxp3^+^ populations in dLN cells is shown. dLN cells, as described in **Figure 7**, were cultured in the absence (-) or presence (+) of 5 µg/mL KLH for 18 hours. Representative plots of OX40^+^CD25^+^ cells from CD4^+^Foxp3^-^ (left) and CD4^+^Foxp3^+^ (right) with (+) or without (-) antigen restimulation are shown. The Δ% value was calculated by subtracting the proportion of OX40^+^CD25^+^ cells without antigen stimulation from the proportion of OX40^+^CD25^+^ cells with antigen stimulation. Data are presented as the mean ± standard deviation (n = 3) from one experiment representative of at least two independent experiments with similar results. *p < 0.05, and ***p < 0.001 indicate statistically significant differences among the groups as determined by the Tukey-Kramer test.

Detection of antigen-responding CD4^+^ T cells is crucial for understanding the outcome of T-cell immunity regulated by the OX40-OX40L system. The AIM assay is used to identify antigen-specific T cells based on the upregulation of activation markers after antigen recall (29, 30). We analyzed Foxp3^-^CD4^+^ and Foxp3^+^CD4^+^ populations in dLN cells from TNP-KLH-immunized mice in terms of the upregulation of the activation markers OX40 and CD25 following stimulation with KLH for 18 hours *in vitro* (**Figure 8**). We found that Fc-scOX40L (C) showed a significantly increased frequency of OX40^+^CD25^+^Foxp3^-^CD4^+^ cells compared to the control (PBS) or the anti-OX40 agonistic antibody (OX86) (**Figure 8**; **Supplementary Figure S6**). Furthermore, Fc-scOX40L (C) exhibited a significantly enhanced frequency of OX40^+^CD25^+^Foxp3^+^CD4^+^ cells (**Figure 8**; **Supplementary Figure S6**). OX40L proteins other than Fc-scOX40L (C) also induced the upregulation of AIM markers, but the levels were marginal (data not shown). These results indicate that an OX40L-fusion protein with an optimized structure may work as a potent agonist for OX40 and that one of the OX40L proteins, Fc-scOX40L (C), allows the expansion of antigen-specific CD4^+^ T cells in the primary phase of the immune response.

**Figure 9:**
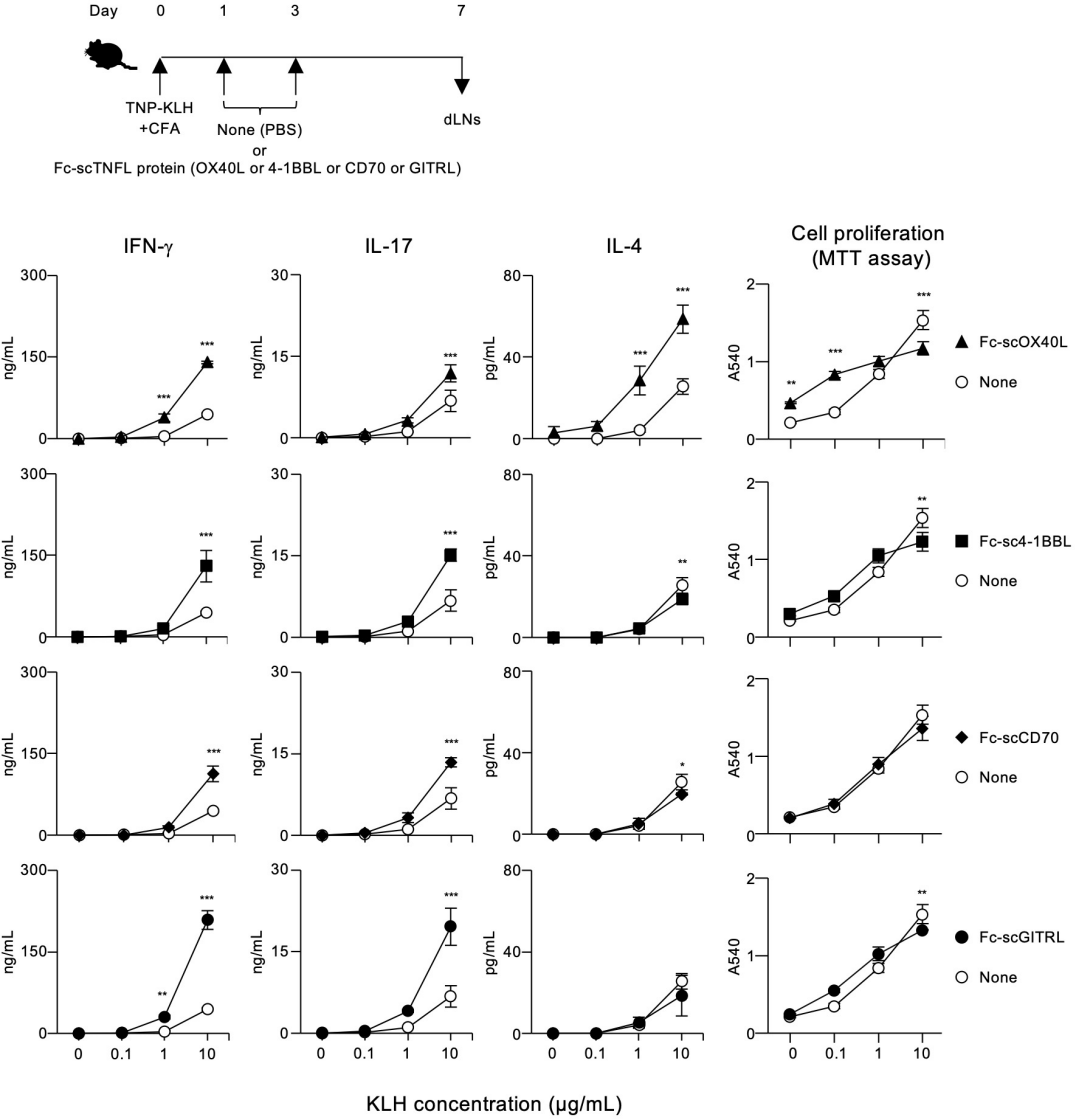
Enhanced antigen-specific effector T cell responses mediated by Fc-scTNFL proteins. The experimental schedule for the induction of antigen-specific T cell responses is shown (upper). C57BL/6 mice were subcutaneously immunized with 100 µg of TNP-KLH emulsified in CFA on day 0, and then intraperitoneally injected twice with 10 µg of the respective Fc-scTNFL proteins, Fc-scOX40L, Fc-sc4-1BBL, Fc-scCD70, and Fc-scGITRL, on days 1 and 3. Popliteal dLNs were harvested on day 7. Pooled dLN cells from three mice per group were cultured with the indicated concentrations of KLH for 3 days. Cell proliferation was evaluated using the MTT assay. Cytokine concentrations were determined by ELISA. Data are presented as the mean ± standard deviation (n = 3) from one experiment representative of at least two independent experiments with similar results. *p < 0.05, **p < 0.01, and ***p < 0.001 indicate significant differences between the Fc-scTNFL-injected group and the control (PBS) group at the respective antigen concentrations (Tukey-Kramer test).

**Figure 10:**
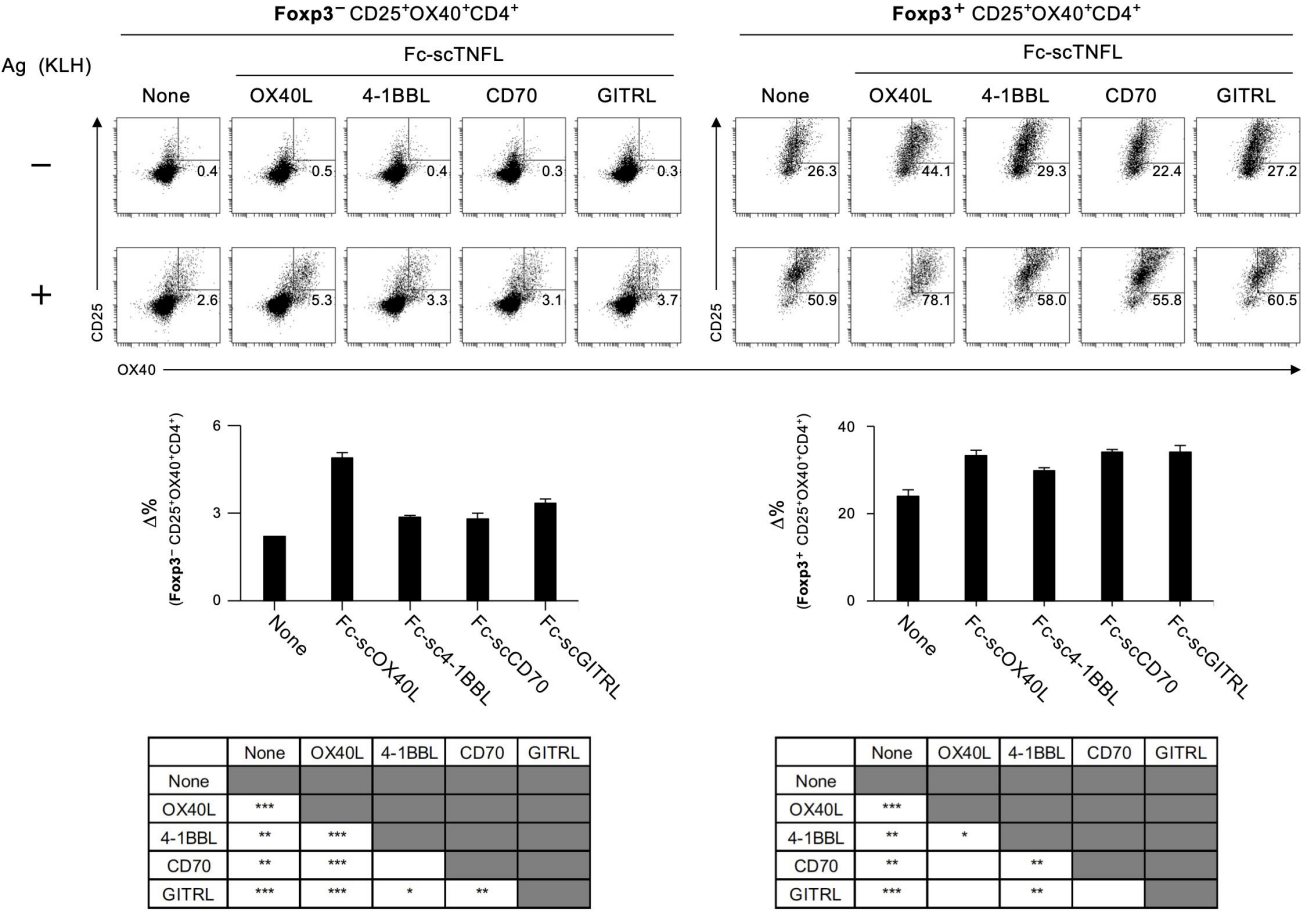
Quantification of antigen-specific CD4^+^ T cell responses mediated by Fc-scTNFL proteins using the AIM assay. The AIM assay was performed as described in **Figure 8**. dLN cells, as in **Figure 9**, were cultured in the absence (-) or presence (+) of 5 µg/mL KLH (+) for 18 hours. Data are presented as the mean ± standard deviation (n = 3) from one experiment representative of at least two independent experiments with similar results. *p < 0.05, **p < 0.01, and ***p < 0.001 indicate statistically significant differences among the groups (Tukey-Kramer test).

The results demonstrated in **Figures 7**, **8**; **Supplementary Figure S6** indicate that a molecule with a structure like Fc-scOX40L (C) would function as a better agonist. To test this hypothesis, we performed similar experiments using molecules in which the OX40L part of Fc-scOX40L (C) was replaced with other TNF ligands (TNFLs), *i.e.*, Fc-sc4-1BBL, Fc-scCD70, and Fc-scGITRL, as we previously reported (24). The TNFL protein moiety was required to induce both proliferative and cytokine responses, and the Fc-only protein could not induce effective T cell responses (**Supplementary Figure S7A**). These Fc-scTNFL proteins significantly enhanced the number of cell divisions in the presence of an anti-CD3 antibody, as determined by the dilution of carboxyfluorescein diacetate succinimidyl ester (CFSE) (**Supplementary Figure S7B**). Mice were immunized with TNP-KLH and CFA, followed by the administration of the respective Fc-scTNFL proteins. Draining lymph node cells from TNP-KLH-immunized mice injected with the respective Fc-scTNFL proteins exhibited higher recall cytokine responses than those treated with the control (PBS) (**Figure 9**). Characteristically, Fc-scOX40L promoted the induction of IL-4-producing cells (**Figures 7**, **9**). In the AIM assay, upon antigen recall, all Fc-scTNFL groups induced the upregulation of the activation markers OX40 and CD25 in both Foxp3^-^CD4^+^ and Foxp3^+^CD4^+^ populations (**Figure 10**). These results suggest that an Fc molecule with scTNFL at the C-terminus can efficiently agonize antigen-primed CD4^+^ T cells *in vivo*.

In summary, our results demonstrate that a potent agonist, capable of enhancing T cell responses *in vivo*, can be developed by incorporating an active single-chain TNF trimer structure into a suitable protein scaffold, such as the Fc domain of an IgG.

## Discussion

OX40 and its ligand OX40L are critical regulators of adaptive immunity. Although OX40 agonism has been explored for immunotherapy, the molecular mechanism underlying its regulation of OX40-mediated T cell responses remains unclear. In this study, five distinct OX40L proteins were designed to evaluate their T cell agonistic activity both *in vitro* and *in vivo*. These OX40L proteins maintained an active structure essential for binding to OX40 and robustly induced OX40-mediated costimulatory signaling in T cells *in vitro*, even in the absence of intentional cross-linking. Administration of one of the OX40L proteins into antigen-immunized mice effectively promoted the expansion of antigen-specific CD4^+^ T cells. Our study provides novel insights into OX40 agonism and the immune regulation mediated by OX40. This information will be valuable for the rational design of novel TNFR agonists for immunotherapy.

An important question is how to generate a soluble OX40L molecule that acts as a potent agonist for OX40. Studies on the generation of OX40L-based OX40 agonists have progressed over the past three decades, focusing on the preparation of OX40L-Fc fusion proteins by linking the extracellular THD of OX40L to the Fc of an IgG (16, 17, 24, 36–43).To stabilize the trimer structure of OX40L, the N-terminal THD of OX40L was connected to a trimer-forming domain, such as the isoleucine zipper coiled-coil domain (16, 43, 44), tenascin-C domain (40), or collagen-like domain (23). Furthermore, a single-chain OX40L molecule, which consists of three copies of the THD of OX40L connected by two linkers on the same polypeptide chain, was produced to evaluate its agonistic activity (24, 42). However, it is still a matter of concern how to create highly active TNF ligand proteins, including OX40L, for practical applications by employing synthetic biology techniques (18, 45–47).

In this study, the collagen-like domain of mannose-binding lectin, a multimerization domain forming trimers or oligomers, and the Fc domain of IgG, a stable scaffold forming dimers, were linked either separately or simultaneously to OX40L to generate five types of OX40L proteins. All these OX40L molecules bound to OX40 and enhanced cytokine production from T cells *in vitro*. Importantly, the administration of one of the OX40L proteins, Fc-scOX40L, which contains the single-chain OX40L at the C-terminus of the Fc domain, effectively promoted the expansion of antigen-specific CD4^+^ T cells. Similar activity was observed in other TNF ligand molecules with a similar structural format. These results suggest that the intrinsic agonistic activity of OX40L can be optimized by engineering the oligomeric architecture of OX40L and that Fc-scOX40L acts as a better agonist for OX40.

To characterize how OX40L proteins augment antigen-specific CD4^+^ T cell responses, the AIM assay (29, 30) was performed based on the upregulation of CD25 and OX40 in the Foxp3-negative CD4^+^ T cell population. Upon antigen restimulation *in vitro*, the frequency of CD25^+^OX40^+^ cells in draining lymph nodes significantly increased within a short period, confirming that this assay is a sensitive method to enumerate antigen-specific CD4^+^ T cells. We found that combining another activation marker, CD69, could be used to detect antigen-specific CD4^+^ T cells (data not shown). From this AIM assay and recall cytokine responses, we found that mice treated with a limited amount of Fc-scOX40L showed a significant increase in conventional effector CD4^+^ T cells compared to the OX86 monoclonal antibody, a benchmark agonistic antibody for OX40. Furthermore, Fc-sc4-1BBL, Fc-scCD70, and Fc-scGITRL also significantly expanded antigen-specific CD4^+^ T cells, demonstrating the potent costimulatory activities of these TNFR agonists. Curiously, in the AIM assay, activation markers were also upregulated in the Foxp3-positive populations. This may be related to the bystander activation of Foxp3^+^ regulatory T cells *in vitro*. It is unclear why the Fc-scTNFL-treated groups exhibited such a phenotype. It is tempting to speculate that OX40L and other TNF ligand proteins used in this study may have a regulatory function for peripherally induced regulatory T cells.

Fc-scOX40L demonstrated superior *in vivo* activity compared to other OX40L fusion proteins. However, the specific structural elements responsible for OX40 agonism, particularly *in vivo*, remain unclear. By incorporating multimerization domains into the THD of OX40L, we successfully generated five distinct types of OX40L fusion proteins. All of these proteins were able to bind to OX40, activate NF-κB, and enhance OX40 costimulation-dependent cytokine production *in vitro*. Nevertheless, their *in vivo* activity, particularly regarding T cell responses, differed from their *in vitro* activity. The MBL domain used in our previous study oligomerizes the OX40L structure, which is essential for OX40 agonism and facilitates the assembly of higher molecular weight protein structures, potentially extending their retention time in the body. Both native full-length mannose-binding lectin in the blood and recombinant mannose-binding lectin have been shown to form oligomeric structures of various molecular sizes (32–34). This MBL domain was incorporated into MBL-OX40L, Fc-MBL-OX40L, and MBL-scOX40L. Additionally, Fc-MBL-OX40L contains a stable IgG1 Fc domain, which could potentially enhance the *in vivo* half-life of the protein. Unexpectedly, we observed reduced *in vivo* activity in these MBL-containing proteins compared to Fc-scOX40L. This finding suggests that forming high molecular weight oligomeric structures alone is insufficient for *in vivo* agonistic activity and that simply fusing OX40L with the IgG1 Fc domain does not enhance its *in vivo* agonistic effect. Furthermore, the hinge region is susceptible to proteolytic cleavage (48–50), indicating that linking OX40L to the N-terminus of the hinge may be disadvantageous. Therefore, further optimization, stabilization, and integration of structural domains are required to maximize OX40 agonism for practical applications.

OX40L-fusion proteins can be optimized for use in human applications. The THD moiety of the mouse OX40L sequence can be replaced with that of human OX40L, which is critical for reducing the immunogenicity of the protein. The human IgG1 Fc moiety in OX40L-fusion proteins can mediate immune effector functions such as antibody-dependent cellular cytotoxicity, antibody-dependent cellular phagocytosis, and complement-dependent cytotoxicity. Consequently, the IgG1 Fc moiety can be substituted with other human IgG subclasses (51–54). Amino acid substitutions in the Fc sequences can enhance or diminish Fc effector functions and modify the circulating half-life of the engineered Fc proteins (55–59). Optimizing the structure of the OX40L-fusion proteins will result in more advanced designer proteins suitable for therapeutic interventions in immune-mediated diseases.

TNF ligand molecules, including OX40L, 4-1BBL, CD70, and GITRL, are all upregulated in activated antigen-presenting cells and promote the activation, differentiation, and survival of effector T cells (1, 60). However, it is uncertain whether these TNF ligands contribute equally to adaptive immunity by quantitatively regulating T cell responses, or by inducing qualitatively different T cell responses, thereby differentially contributing to a specialized immune response. The results suggest that the TNF ligand-fusion proteins quantitatively control T cell responses in the primary immune response. Administration of respective TNF ligand proteins with antigen and adjuvant promoted the expansion of antigen-specific T cells producing effector cytokines. On the other hand, from a qualitative perspective, we observed that Fc-scOX40L specifically promoted IL-4-producing cells. This may be related to OX40’s role in inducing Th2-type immune responses. OX40L interactions with OX40^+^ T cells enhance key signals in adaptive type 2 immunity (61–72), making the OX40L-OX40 signaling axis a promising target for intervention in allergic diseases, including atopic dermatitis (9, 73–75). Augmenting antigen-specific T cell responses mediated by TNFR agonists could be beneficial for controlling immunity against infections and cancers (76–82). It is important to further characterize how the unique attributes of the OX40L protein influence *in vivo* T cell responses, including antitumor immunity.

In summary, we demonstrated that the oligomeric status of OX40L critically affects its agonistic activity. Although this information is valuable for designing TNFR agonists, further research is needed to understand the mechanisms governing OX40 agonism for immunotherapy.

## Data Availability

The original contributions presented in the study are included in the article/**Supplementary Material**. Further inquiries can be directed to the corresponding author.
