# Supplementary figures and images for: Generation and characterization of OX40-ligand fusion protein that agonizes OX40 on T-Lymphocytes
Source: Front Immunol. 2025 Jan 10;15:1473815. doi: 10.3389/fimmu.2024.1473815 (PMC11757143; doi:10.3389/fimmu.2024.1473815)

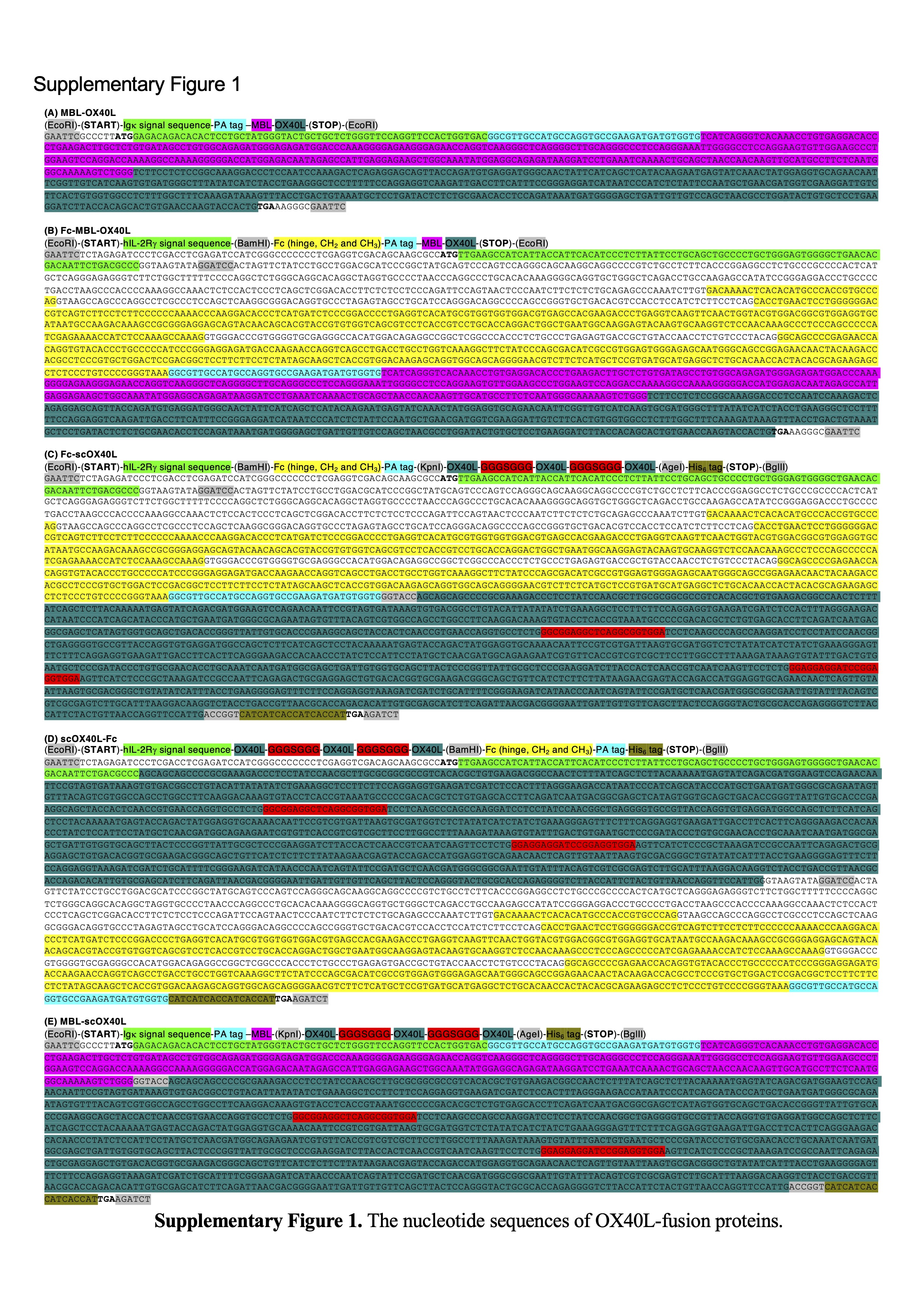

Supplement: Supplementary file 1 [file Image1.jpeg]

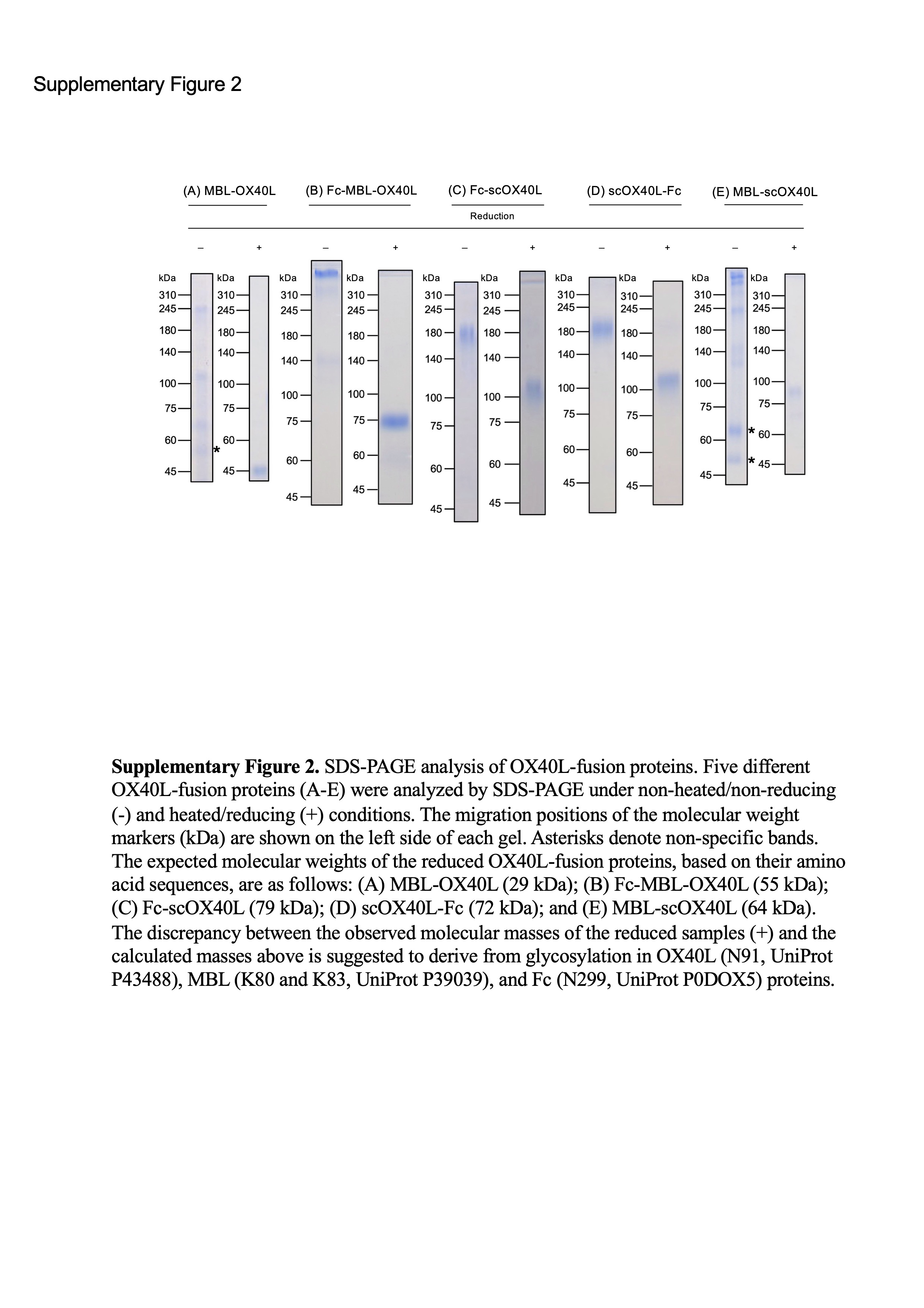

Supplement: Supplementary file 2 [file Image2.jpeg]

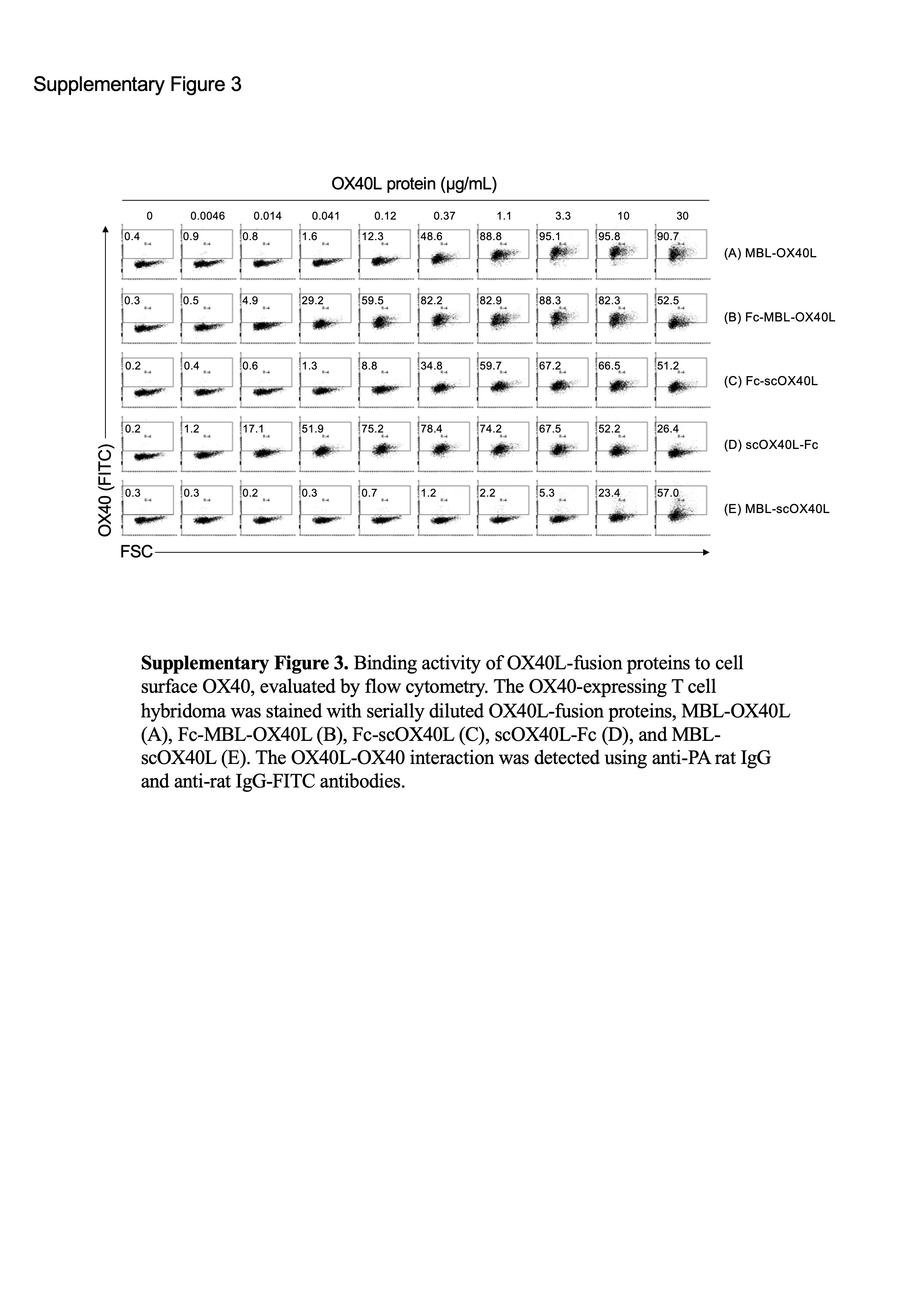

Supplement: Supplementary file 3 [file Image3.jpeg]

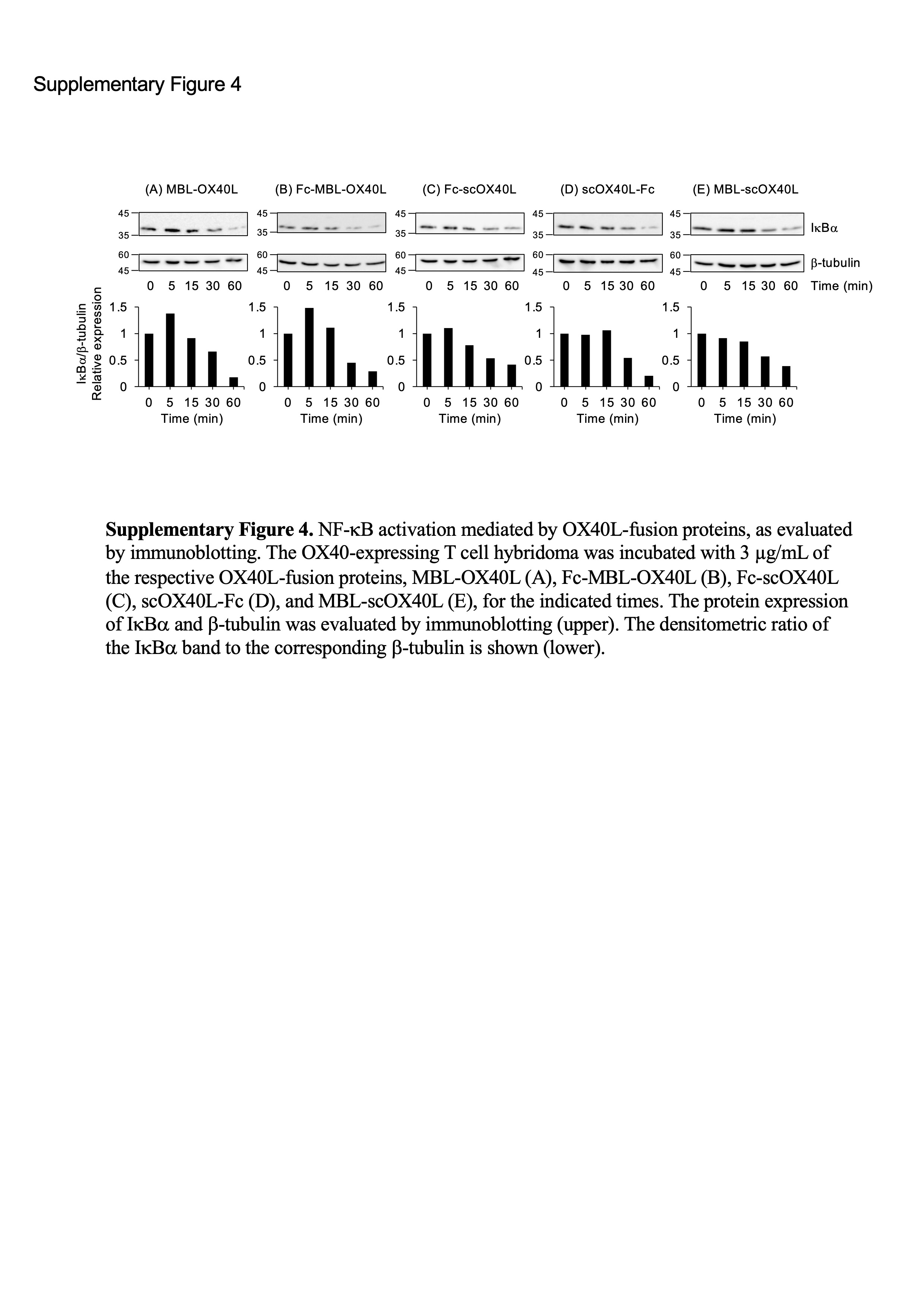

Supplement: Supplementary file 4 [file Image4.jpeg]

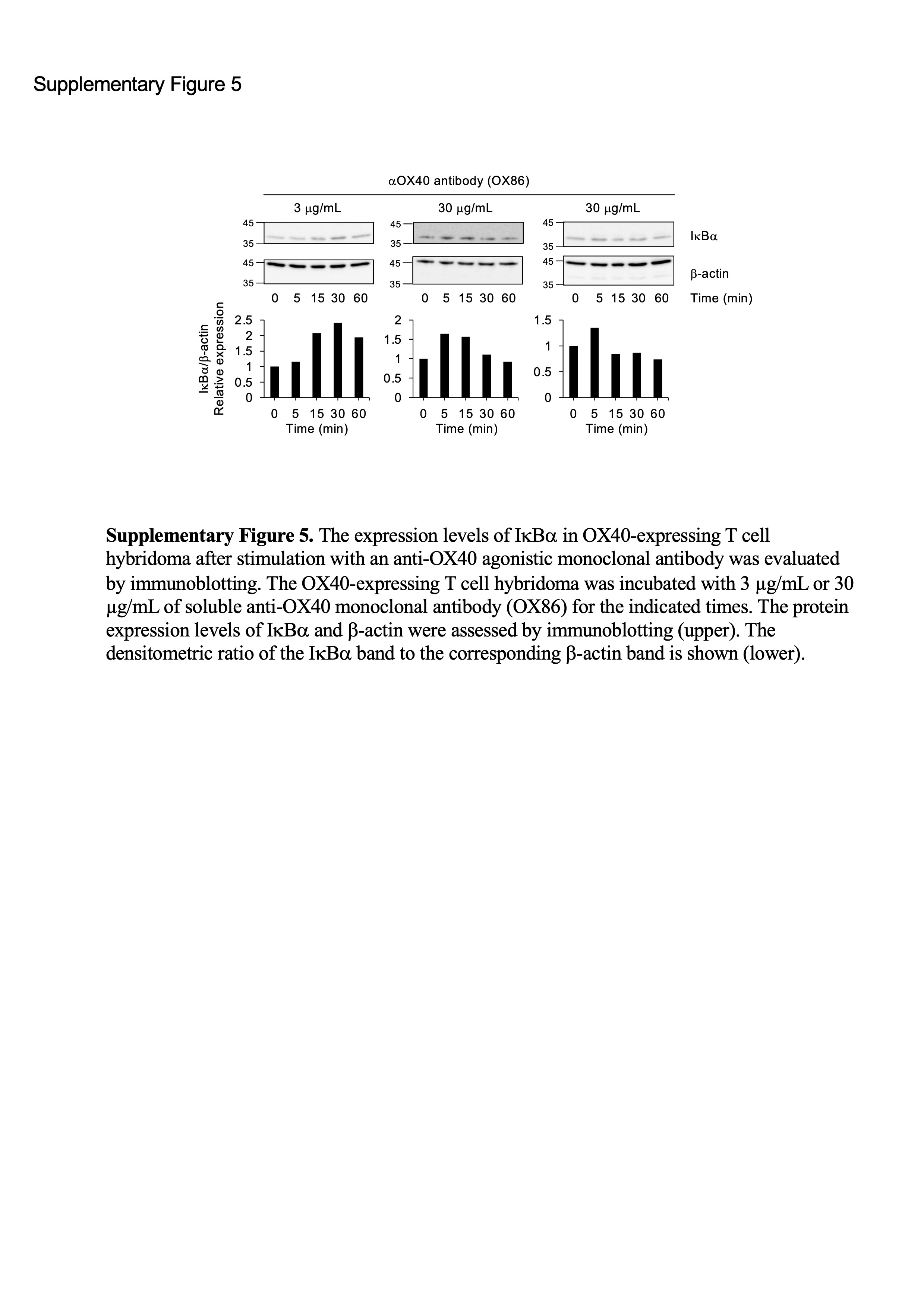

Supplement: Supplementary file 5 [file Image5.jpeg]

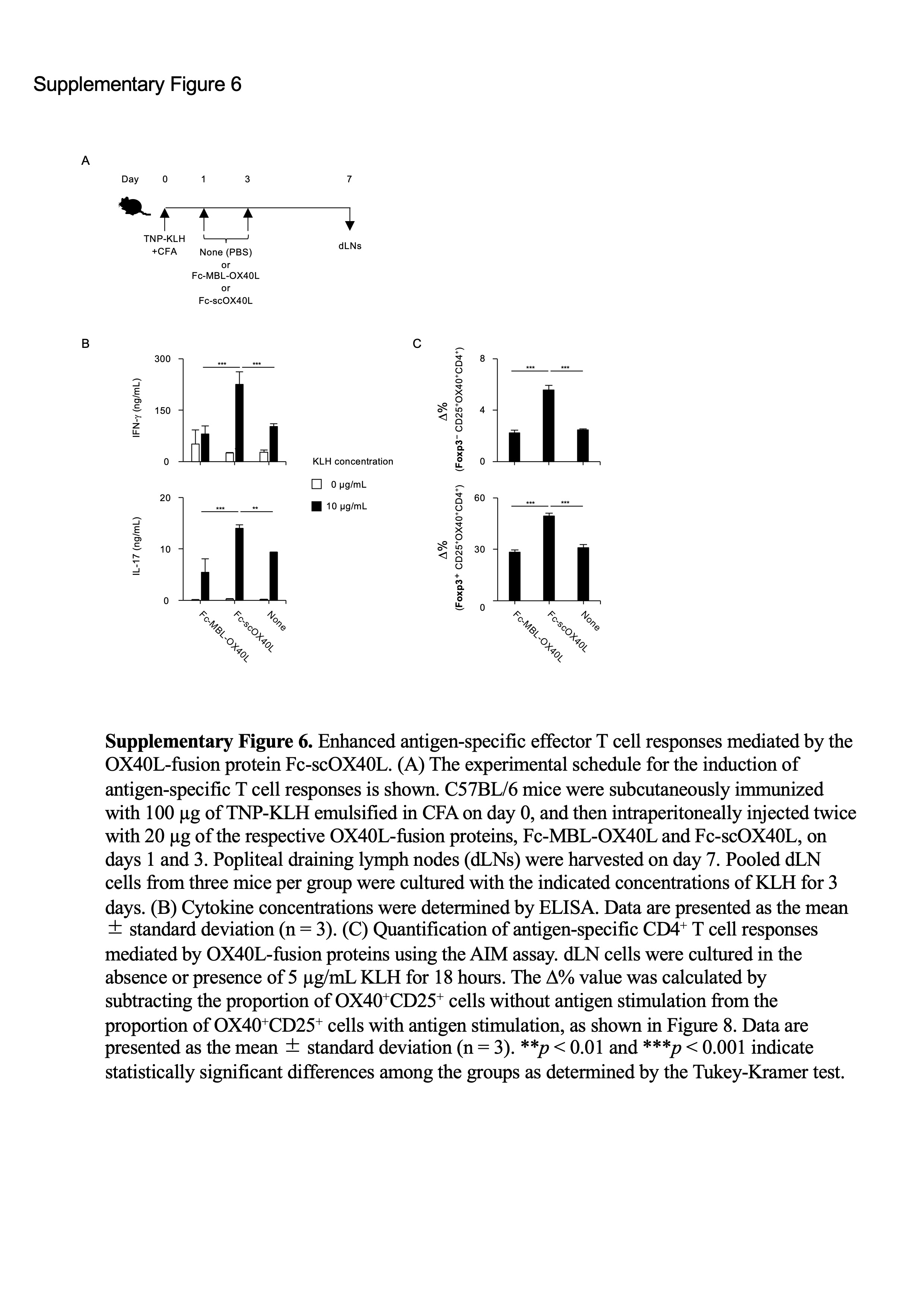

Supplement: Supplementary file 6 [file Image6.jpeg]

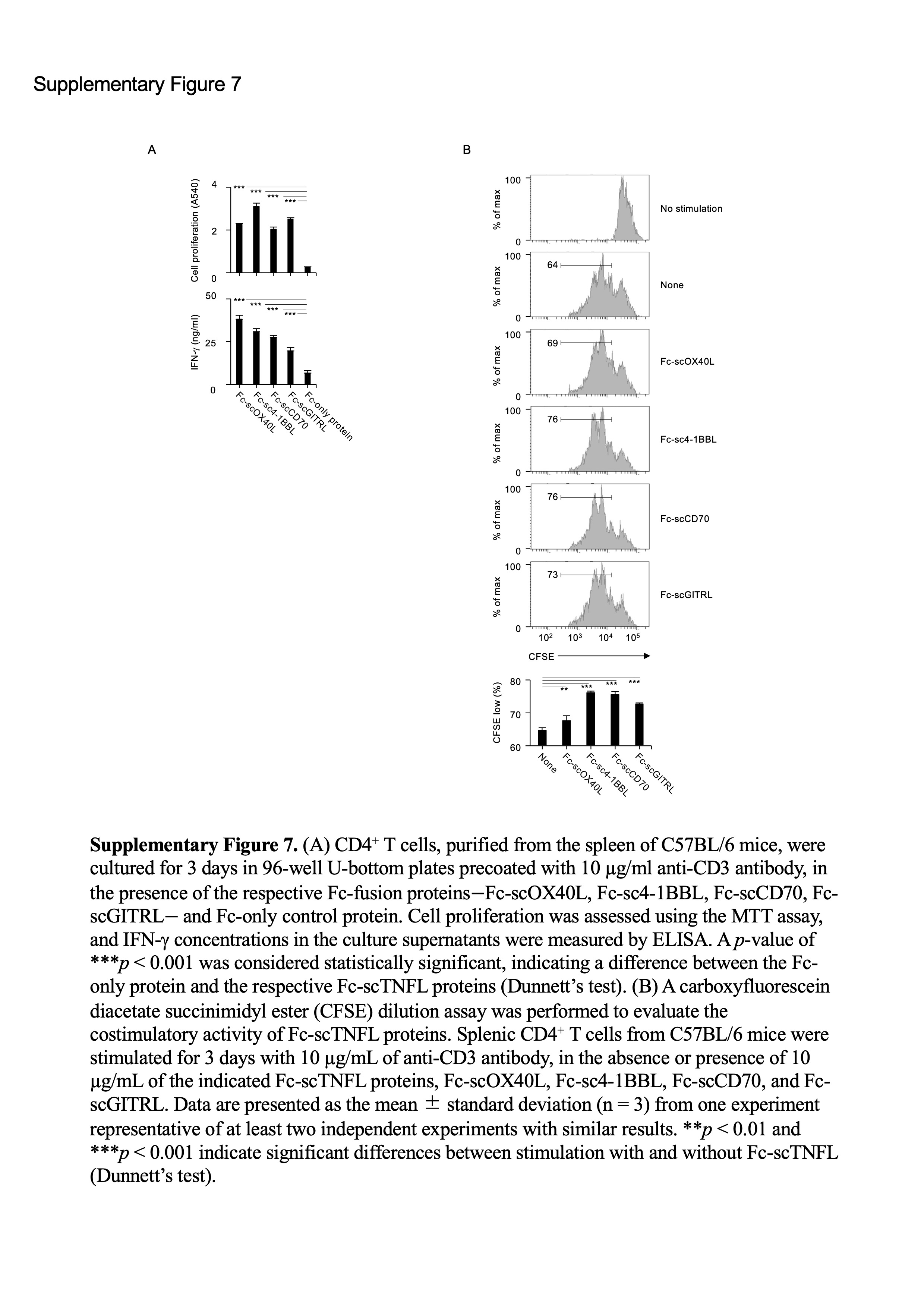

Supplement: Supplementary file 7 [file Image7.jpeg]
